# The effects of aquatic interventions on emotional and behavioral outcomes in children with autism spectrum disorder

**DOI:** 10.3389/fpsyg.2026.1929354

**Published:** 2026-09-23

**Authors:** Miao Zhao, Cheng Chen, Yupeng Shen, Jiji Zhao, Jiayi Sun

**Affiliations:** 1 School of Physical Education & Sports Science, South China Normal University, Guangzhou, Guangdong, China; 2 Sports Department, Guangzhou Medical University, Guangzhou, Guangdong, China; 3 Guangzhou Huangpu District Qimingxing School, Guangzhou, Guangdong, China; 4 Sports Academy, Guangzhou College of Commerce, Guangzhou, China

**Keywords:** aquatic intervention, autism spectrum disorder, evidence-based practice, sensory regulation, systematic review, water-based therapy

## Abstract

Children with autism spectrum disorder (ASD) commonly experience emotional and behavioral difficulties that may arise from atypical sensory processing, and the sensory properties of water closely correspond to these characteristics. We systematically evaluated the effects of aquatic interventions on four pre-specified outcome domains—emotion-related functioning, social-emotional functioning, behavioral outcomes, and quality of life—in children with ASD. Eight bibliographic databases and one trial registry were searched from inception to July 2026 using a two-concept strategy without language restriction, for studies involving children with ASD who received a structured aquatic intervention and reported a validated outcome. Methodological quality was assessed using RoB 2, ROBINS-I, and single-case design criteria, and the certainty of evidence was evaluated with GRADE. Owing to heterogeneous outcome instruments and the small number of controlled studies, a meta-analysis was not conducted; in accordance with the Synthesis Without Meta-analysis (SWiM) guideline, findings were synthesized narratively by domain, with the direction of effect and statistical significance tabulated and illustrated in an effect-direction (harvest) plot. Nineteen publications, describing up to 18 independent studies (approximately 320 participants in total from 10 countries, including non-ASD comparison participants; the number of unique participants with ASD is uncertain), with diverse designs and varying methodological quality were included, most of which were uncontrolled and had a high risk of bias. Across all four domains, the direction of effect supported aquatic intervention, and no study showed an effect favoring the comparator. The evidence was most concentrated and internally consistent for behavioral outcomes, for which all six controlled studies favored aquatic intervention, and five reported statistically significant results—although this tally reflects direction only and should not be equated with demonstrated effectiveness, whereas findings for social-emotional, emotion-related, and quality-of-life outcomes were positive in direction but mostly non-significant. Only one trial included an active land-based comparator, so a water-specific effect could not be distinguished. The available evidence suggests a consistent favorable direction across emotional and behavioral domains in children with ASD, but the evidence remains limited, heterogeneous, and at high risk of bias; sufficiently powered trials using active comparators and validated common outcome measures are needed.

**Systematic review registration:**

PROSPERO: https://www.crd.york.ac.uk/prospero/display_record.php?ID=CRD420261390044, Identifier: CRD420261390044.

## 1 Introduction

Autism spectrum disorder (ASD) is a neurodevelopmental condition defined by persistent impairments in social communication and interaction, along with restricted and repetitive patterns of behavior, interests, or activities (American Psychiatric Association, 2013). In line with the continued increase in global prevalence over the past two decades (Zeidan et al., 2022), about one in 36 children in the United States is identified as having ASD according to the CDC Autism and Developmental Disabilities Monitoring Network (Maenner et al., 2023). In addition to the core diagnostic features, children with ASD often show co-occurring emotional and behavioral difficulties that are major contributors to family stress, mental-health-service use, and impaired quality of life, with substantial effects on daily functioning, family dynamics, and long-term development. Emotion dysregulation has been reported in up to 80% of children with ASD, contributing to higher rates of anxiety, depression, and externalizing problems such as aggression and irritability (Mazefsky et al., 2013; Samson et al., 2015), while restricted and repetitive behaviors—a central feature of ASD—often increase under sensory overload or emotional distress, producing a self-reinforcing cycle that further compromises adaptive behavior and social functioning (Boyd et al., 2010). These difficulties are strongly related to poorer quality of life and are increasingly viewed as intervention targets in their own right rather than only as consequences of the core features. Importantly, emerging evidence suggests that these problems may share a common underlying pathway based on atypical sensory processing and regulation (Mazefsky and White, 2014).

Because these difficulties are proposed to share a substrate of atypical sensory processing and regulation, the same framework guides the outcomes considered in this review. The four pre-specified domains are better understood not as separate endpoints but as sequential downstream expressions of one regulatory mechanism, arranged along a proximal-to-distal gradient. Emotion-related functioning would be the most proximal domain, on the assumption that poor modulation of physiological arousal gives rise to emotional reactivity, anxiety, and internalizing and externalizing symptoms most directly associated with autonomic state. Behavioral outcomes—especially stereotyped and repetitive behaviors—have been interpreted as a regulatory response to such dysregulation that may help modulate arousal during sensory overload (Boyd et al., 2010). Social-emotional functioning would be more distal and integrated, if persistent hyperarousal promotes social withdrawal such that participation depends on a previously regulated state; quality of life would represent the most distal and cumulative outcome. Each of these proposed links is an assumption of the framework rather than an established finding. Examining these four domains separately therefore covers the full causal span of the proposed mechanism and enables direct assessment of its central prediction: that an intervention targeting the sensory substrate should have the strongest and most reliable effects on proximal domains and weaker, more variable effects on distal domains. This gradient is treated as a hypothesis to be tested rather than assumed, acknowledging that uneven study availability restricts the extent to which any observed pattern can confirm it, whereas a single pooled estimate would obscure this structure.

Aquatic interventions—including structured swimming, hydrotherapy approaches such as the Halliwick method and Watsu, multisystem aquatic therapy, and water-based exercise—have gained increasing attention for children with ASD, based on a sensory-regulation rationale: the distinctive sensory features of the water environment may directly address the sensory-regulation deficits underlying these emotional and behavioral difficulties. Buoyancy decreases gravitational load and proprioceptive demand, which has been proposed to reduce physiological arousal and anxiety (Becker, 2009); hydrostatic pressure delivers continuous, uniform deep-pressure input that may activate the parasympathetic nervous system and has been hypothesized to support calming and reduce emotional reactivity and externalizing behavior (Mooventhan and Nivethitha, 2014); water maintained at about 30–34 °C has been reported to lower sympathetic tone and to promote psychological wellbeing (Haghayegh et al., 2019); and the rhythmic, repetitive quality of aquatic movement offers structured proprioceptive and vestibular input that may regulate the sensory thresholds involved in stereotypic and repetitive behavior (Boyd et al., 2010). The organized social setting of group-based aquatic programs also offers naturalistic opportunities for social interaction. Consistent with a parasympathetic mechanism, Bell reported a significant rise in vagal tone in children with ASD after an aquatic session (Bell, 2021). Overall, the water environment may operate as a naturalistic sensory-regulation context that simultaneously addresses emotion regulation, social-emotional functioning, behavioral regulation, and general wellbeing through an integrated sensory mechanism.

An increasing number of primary studies have examined aquatic programs for emotional and behavioral outcomes in children with ASD. However, existing systematic reviews have mainly emphasized motor-skill development and gross-motor outcomes: one recent review synthesized 19 aquatic-intervention studies but restricted its analysis to motor and social outcomes (van t Hooft et al., 2024), another assessed aquatic effects on physical fitness and aquatic skills (Aleksandrovic et al., 2016), and a recent meta-analysis examined physical-activity interventions for repetitive behaviors but was not specific to aquatic environments (Yang and Li, 2025). An earlier narrative review of the social and behavioral aspects of hydrotherapy did not undertake a meta-analysis because the available data were insufficient (Mortimer et al., 2014). To our knowledge, the present review is the first prospectively registered systematic review to examine aquatic interventions across four functionally distinct emotion- and behavior-related domains using structured synthesis-without-meta-analysis methods; the first to foreground the active-comparator problem by distinguishing what the current evidence can establish about a water-specific effect from the effect of structured activity more broadly; and the first to interpret these outcomes through a sensory-regulation lens, which is advanced here as an organizing hypothesis rather than an established framework.

We therefore conducted a systematic review—prospectively registered (PROSPERO: CRD420261390044) and reported according to the PRISMA 2020 statement (Page et al., 2021)—to identify, evaluate, and synthesize the available evidence on the effects of aquatic interventions on emotional and behavioral outcomes in children and adolescents with ASD, covering four pre-specified outcome domains: (a) emotion-related functioning; (b) social-emotional functioning; (c) behavioral outcomes; and (*d*) quality of life and wellbeing. A secondary aim was to determine whether the effects were moderated by intervention type, intervention duration, participant age, or ASD severity. Beyond estimating effects within each domain, a key objective was to describe how the quantity, quality, and reliability of the evidence varied across domains—that is, which outcomes the current evidence can, and cannot, support with confidence. Because the field was expected to consist mainly of uncontrolled designs and heterogeneous outcome measures, a structured narrative synthesis arranged by outcome domain was selected as the primary, pre-specified synthesis method; the registered meta-analysis was not performed once the included evidence was found to be too limited and too heterogeneous to provide a comparable pooled estimand (see Section 2).

## 2 Materials and methods

### 2.1 Search strategy

This systematic review was performed and reported in accordance with the PRISMA 2020 statement (Page et al., 2021) and was prospectively registered with PROSPERO (CRD420261390044). PubMed, Embase, Web of Science, PsycINFO, CINAHL, and the Cochrane Library (including CENTRAL), together with the ClinicalTrials.gov trial registry and the reference lists of relevant reviews, were searched from inception to 15 May 2026, without restriction on publication year. In the initial search, terms were organized into three concept blocks (population, intervention, and outcome) and combined using Boolean logic. To maximize sensitivity, the search was subsequently updated using a two-concept strategy (population and intervention only, without outcome restrictions), with expanded intervention vocabulary (including Halliwick, Watsu, and adapted aquatics) and two additional databases (SPORTDiscus and ERIC), as well as forward and backward citation tracking of all included studies; the complete database-specific search strategies are provided in Supplementary Appendix S1. The updated search, executed in July 2026 without language restriction, did not identify any additional eligible studies beyond the original 19 among the reports that could be retrieved and evaluated. Two reviewers independently conducted the search according to the eligibility criteria, and disagreements were resolved through discussion.

### 2.2 Eligibility criteria

Eligibility criteria were defined according to the PICOS framework and are summarized in Table 1.

**Table 1:** Eligibility criteria (PIC mOS).

Parameter	Definition
Population	Children and adolescents ( ≤ 17 years) with a formal clinical diagnosis of ASD, made according to the diagnostic standards in use at the time of the study (DSM-IV, DSM-5, or ICD-10) or established using a validated diagnostic instrument, with no restrictions on severity, intellectual ability, or comorbidity.
Intervention	Any structured aquatic intervention delivered in water, including structured swimming, Halliwick, Watsu, multisystem aquatic therapy, aquatic exercise, or aquatic occupational therapy, with a defined protocol. Dolphin-assisted therapy, virtual aquatic environments, and non-separable multicomponent interventions were excluded.
Comparator	Any comparator, including no intervention/waitlist, treatment as usual, land-based exercise, other active interventions, or no comparator; single-group and single-case designs were eligible.
Outcomes	At least one validated outcome in one of four pre-specified outcome domains: emotion-related functioning, social-emotional functioning, behavioral outcomes, and quality of life. Additional outcomes included adverse events, satisfaction, adherence, and dropout.
Study design	RCTs, quasi-experimental/controlled clinical trials, single-group pre–post designs, SCEDs (e.g., ABAB or multiple baseline designs) with ≥3 within-subject demonstrations of effect across behaviors, settings, or baselines, irrespective of participant number; case series (*n* ≥ 3); uncontrolled case reports lacking within-subject replication were included for qualitative synthesis only;.

ASD, autism spectrum disorder; DSM-5, Diagnostic and Statistical Manual of Mental Disorders, 5th edition; ICD-10, International Classification of Diseases, 10th revision; PICOS, Population, Intervention, Comparator, Outcome, Study design. The four outcome domains and their instruments were pre-specified in the registered protocol.

**Types of studies**. Reports published in peer-reviewed journals were considered eligible. Eligible designs included randomized controlled trials (RCTs), non-randomized or quasi-experimental controlled trials, single-group pre-post studies, and single-case experimental designs (SCEDs; e.g., ABAB or multiple-baseline designs). Because SCEDs establish experimental control through the replication of effects within participants—across multiple behaviors, settings, or baseline lengths—rather than through participant number, SCEDs with fewer than three participants were eligible if the design provided at least three demonstrations of an effect; uncontrolled case reports without such within-subject replication were included for qualitative description only. Systematic or narrative reviews, editorials, conference abstracts without extractable data, and reports from which valid outcome data could not be retrieved were excluded.

**Types of participants**. Children and adolescents ( ≤ 17 years) with a formal clinical diagnosis of ASD were eligible. In applying this criterion, a diagnosis was accepted when it was made according to the diagnostic standards in use at the time of the study (DSM-IV, DSM-5, or ICD-10) or was established using a validated diagnostic instrument. This operationalization is broader than the wording of the registered protocol, which referred to DSM-5, ICD-10, or a validated instrument; the deviation was introduced because several older included studies predated DSM-5 and reported DSM-IV-based or clinician-made diagnoses without naming a standardized instrument. Where a study did not report the diagnostic basis (denoted NR), the study was retained only when the original report stated that participants had a formal ASD diagnosis; NR was not itself interpreted as evidence that the criterion was satisfied. No restrictions were applied regarding sex, intellectual ability, ASD severity, or comorbidity.

**Types of interventions**. The experimental group received a structured aquatic intervention delivered in water (structured swimming, the Halliwick concept, Watsu, multisystem aquatic therapy, aquatic exercise, or aquatic occupational therapy) with a defined protocol; comparators included no intervention or waitlist, treatment as usual, an active land-based or other intervention, or none (for single-group and single-case designs). Dolphin-assisted therapy, virtual aquatic environments, and non-separable multi-component interventions were excluded.

**Types of outcome measures**. Studies were required to report at least one outcome assessed with a validated instrument in one of four pre-specified domains: emotion-related functioning, social-emotional functioning, behavioral outcomes, or quality of life. Adverse events, satisfaction, and adherence or dropout were recorded as supplementary outcomes. Reports that used only non-validated, author-constructed instruments were retained for narrative description and were not included in any quantitative analysis.

### 2.3 Study selection and data extraction

Two reviewers independently screened titles and abstracts and subsequently assessed the full texts of potentially eligible records against the eligibility criteria, with decisions cross-checked between reviewers; disagreements were settled by discussion or, when needed, by a third reviewer. Title and abstract screening was supported by ASReview active-learning software (Van De Schoot et al., 2021), although all final inclusion decisions were made by the reviewers (Cohen's κ = 0.78). Records were managed in a reference manager, and duplicate entries were removed. Using a piloted extraction form, two reviewers independently extracted and cross-checked the following information: (1) study identifiers (first author, year, and country); (2) study design; (3) participant characteristics (sample size, age, sex, and basis of ASD diagnosis); (4) intervention and comparator details (type, session length, frequency, and total duration); (5) outcome domains, instruments, and measurement time points; and (6) quantitative findings (means, standard deviations, effect sizes, and test statistics), all verified against the primary reports. When a study reported subscales covering more than one domain, each subscale was independently allocated to its relevant domain by the two reviewers (Cohen's κ = 0.84).

### 2.4 Risk of bias and certainty assessment

Methodological quality was independently evaluated by two reviewers, with disagreements resolved by discussion or by a third reviewer. RCTs were assessed using the Cochrane Risk-of-Bias tool (RoB 2; Sterne et al., 2019), non-randomized and quasi-experimental studies using ROBINS-I (Sterne et al., 2016), and single-case experimental designs using design-specific risk-of-bias standards (What Works Clearinghouse, 2020); each study was assigned an overall judgment of low, moderate (some concerns), or high risk of bias. Because these three tools assess different sources of bias and use different judgment categories, the assessments are reported separately by tool: outcome-specific RoB 2 judgments across the five original domains for the randomized trials (Supplementary Table S2), and ROBINS-I or single-case design criteria for the remaining studies (Supplementary Table S3). Figure 1 presents these assessments in separate panels by assessment framework, as a summary visualization only, and domain-level or overall judgments derived from different tools should not be treated as directly interchangeable. The certainty of the evidence body for each domain was then assessed using the GRADE approach (Guyatt et al., 2008) as high, moderate, low, or very low, with downgrading applied for risk of bias, inconsistency, indirectness, imprecision, and publication bias.

**Figure 1:**
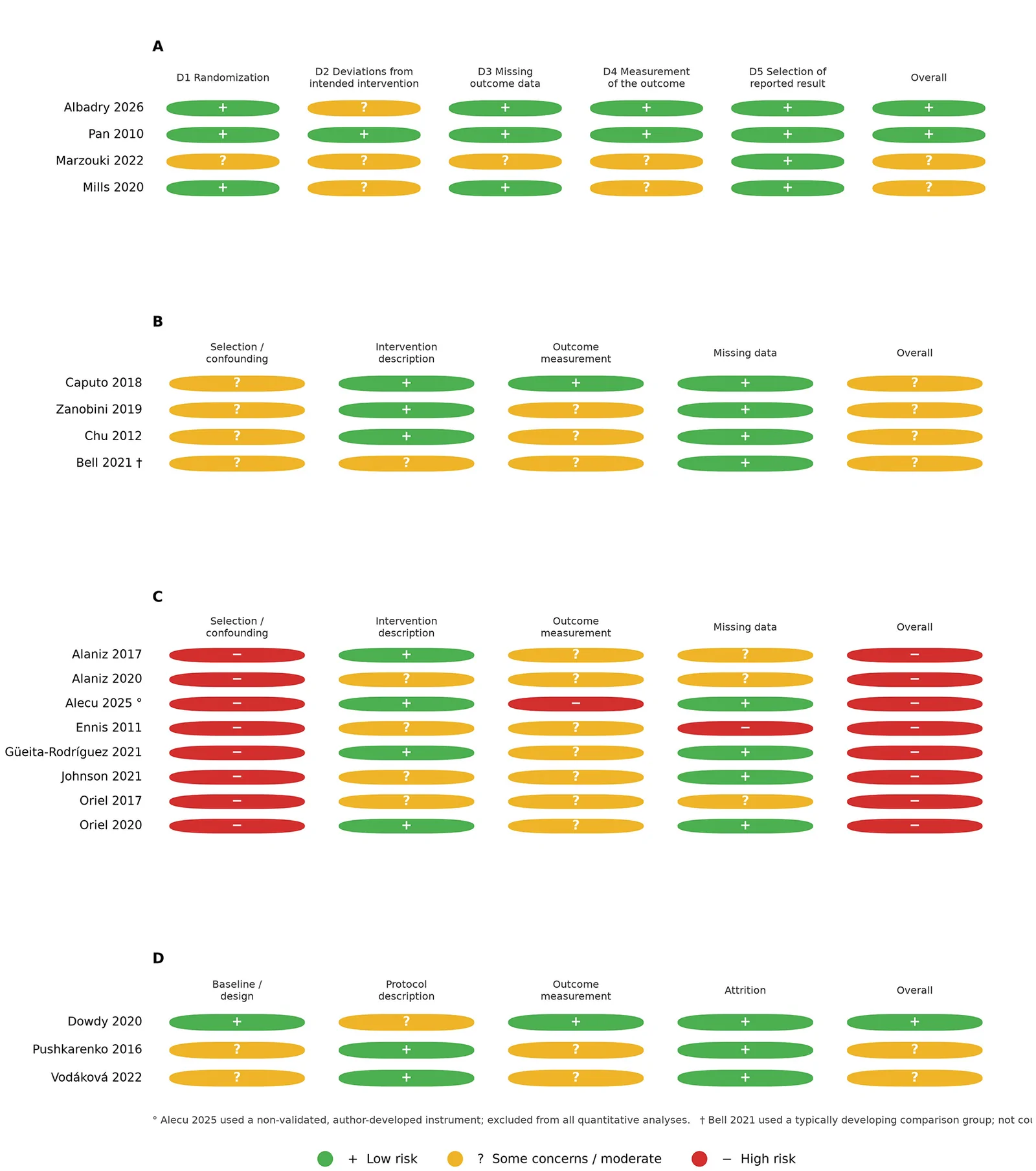
Risk-of-bias summary across the 19 included studies, displayed in separate panels by assessment framework: RoB 2 (randomized trials, five original domains), ROBINS-I (controlled non-randomized studies), adapted criteria (single-group pre–post studies), and single-case experimental design standards. Panels use each tool's native domains and are not directly comparable across frameworks; the tool-specific assessments in Supplementary Tables S2, S3 are the authoritative presentation. **(A)** Randomized controlled trials—Cochrane RoB 2. **(B)** Non-randomized studies assessed with ROBINS-I. **(C)** Single-group pre-post studies—adapted criteria. **(D)** Single-case experimental designs—SCED standards (WWC). Green (+) = low risk; amber (?) = some concerns/moderate risk; red (–) = high/serious risk, using the categories native to each framework. Judgments are not transferable between panels.

### 2.5 Data synthesis

A meta-analysis was not performed because the outcome measures were highly heterogeneous and the number of controlled studies was insufficient to generate a comparable pooled estimand. Specifically, the sources of heterogeneity that precluded quantitative pooling included: differences in study design (parallel-group and crossover RCTs, non-randomized controlled studies, single-group pre-post studies, and single-case experimental designs); different comparator types (no intervention, usual care, active land-based exercise, and typically developing children); inconsistent outcome definitions and instruments (more than 15 different measures across 19 studies, with no single instrument used in a directly comparable form across three or more controlled studies); variation in intervention type, dose, and duration (ranging from a single session to 40 weeks); and variation in the timing of outcome assessments. In accordance with the Synthesis Without Meta-analysis (SWiM) guideline (Campbell et al., 2020), the findings were synthesized narratively and organized according to outcome domain. For each study, the direction of effect (favoring the aquatic intervention, indicating no clear difference, or favoring the comparator) together with its statistical significance was extracted, summarized in tables, and presented in an effect-direction (harvest) plot, with study design and overall risk of bias also indicated; effect sizes reported by individual studies were interpreted descriptively using Cohen's criteria (small ≈ 0.2, medium ≈ 0.5, large ≈ 0.8). Where the reported data permitted, study-level standardized effect sizes with 95% confidence intervals were extracted or computed for every controlled comparison and are displayed without a pooled estimate in Supplementary Figure S1; uncontrolled pre-post estimates are shown separately and identified as such. Effect sizes were computed as standardized mean differences (Cohen's *d*). No small-sample bias correction (Hedges' adjustment) was applied: because the estimates are displayed descriptively, without pooling or inverse-variance weighting, the correction would change neither the direction nor the statistical significance of any comparison, and the uncorrected values remain directly comparable with the primary-study reports. Post-intervention means and standard deviations were used where reported; change scores were used only when post-intervention values were unavailable, and the two metrics were never combined within the same comparison. Effects reported as other statistics (*t, F*, or exact *P* values) were converted to standardized mean differences using standard formulae, and 95% confidence intervals were derived from the standard error of *d* using the normal approximation. For crossover trials, within-participant correlation coefficients were not reported; these comparisons were therefore analyzed as if they were parallel-group contrasts of post-intervention values, an approach that yields conservative (wider) confidence intervals. Full computational details, including the formulae used and the data source for each estimate, are provided in Supplementary Methods S6. A mapping of each outcome instrument to its assigned domain is provided in Supplementary Table S5. Because effect-direction (vote-counting) syntheses may be influenced by studies with a high risk of bias, we additionally examined whether the overall favorable direction remained when consideration was limited to randomized and non-randomized controlled studies. The pre-specified moderator and subgroup analyses (intervention type, intervention duration, ASD severity, and participant age) were not undertaken because an insufficient number of studies contributed comparable data. The inability to conduct these analyses limits interpretation of the findings, because it remains unknown whether the observed favorable direction varies by intervention characteristics, participant age, or ASD severity; these moderators should be prioritized in future primary studies designed to allow such comparisons. As no pooled analyses were conducted, funnel plots and Egger's tests for small-study effects were not applicable; instead, the possibility of reporting bias was considered descriptively. Data management, tabulation, and generation of the effect-direction plot were completed using Python 3.11.

## 3 Results

### 3.1 Search results

The study selection process is presented in Figure 2. Database searches of the eight bibliographic databases and the clinical trials registry identified 1,502 records. After removal of 649 duplicate records, 853 records remained for title and abstract screening, of which 719 were excluded. One hundred thirty-four reports were considered potentially eligible for full-text assessment, and 99 full-text articles were successfully retrieved; the remaining 35 reports could not be obtained despite searches of institutional collections, interlibrary-loan requests, and direct correspondence with the study authors (a complete list is provided in Supplementary Table S4). Because this represents just over one quarter of potentially eligible reports, availability bias cannot be excluded, and the potential effect of this missing evidence should be considered when interpreting the findings. Following full-text evaluation of the 99 retrieved articles, 80 reports were excluded. Ultimately, 19 studies satisfied the eligibility criteria and were included in the final synthesis. The record counts above are those of the updated two-concept search across the nine sources, executed in July 2026 and supplemented by forward and backward citation tracking of all included studies; database-specific strategies are given in Supplementary Appendix S1. Two reports from the same research group (Alaniz et al., 2017, 2020) described closely related work and may have included overlapping participants; therefore, they were retained as separate publications but were not allowed to contribute overlapping effect sizes to any pooled analysis.

**Figure 2:**
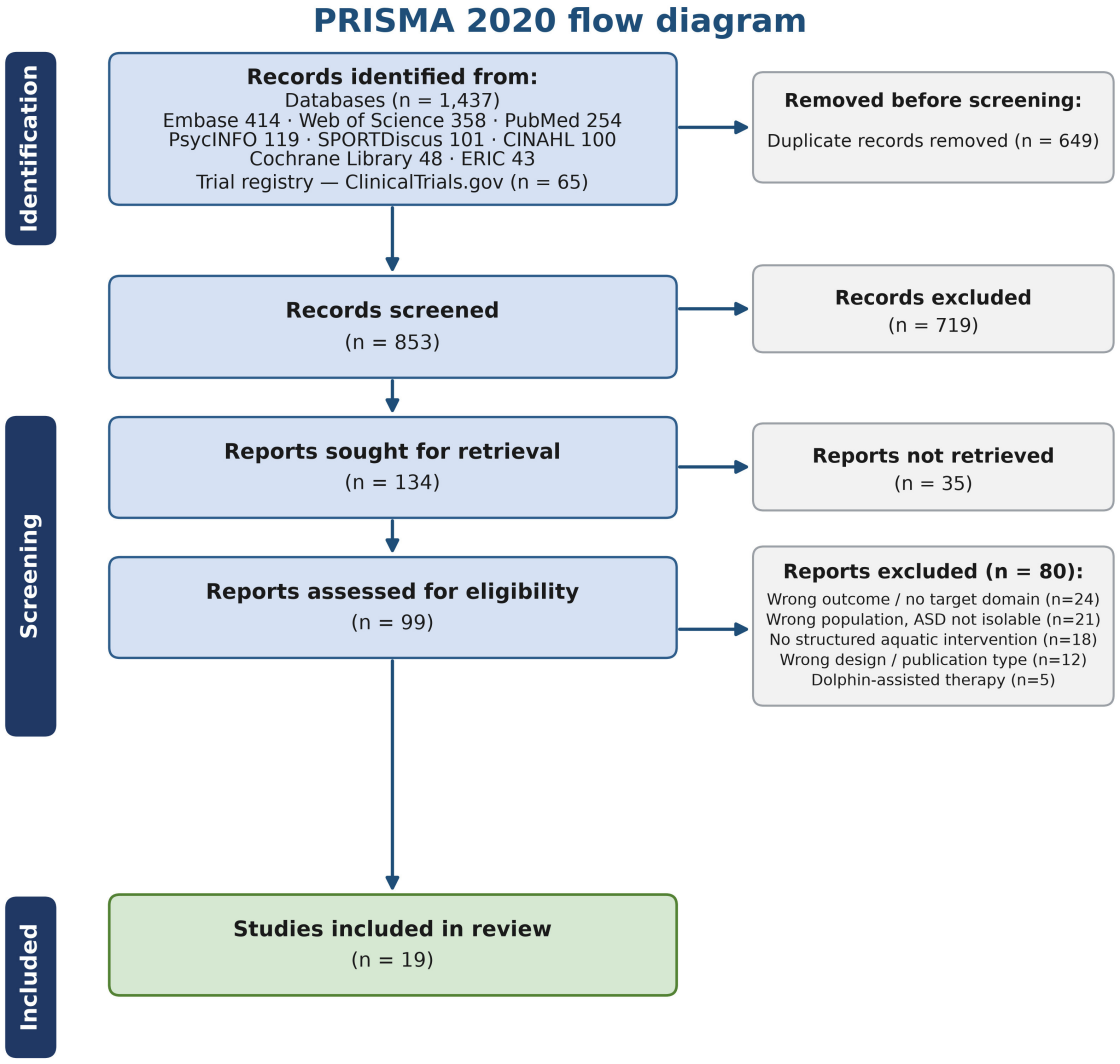
PRISMA 2020 flow diagram of study identification and selection. Exclusion reasons at the full-text stage are presented in this figure.

### 3.2 Methodological quality

Among the four RCTs, two were judged to have a low risk of bias (Albadry et al., 2026; Pan, 2010); in Pan (2010) the randomization procedure was adequate, outcome assessors were blinded to allocation, and all randomized participants completed the study, although the crossover design carries a risk of carryover that is addressed in the Discussion. The remaining two were rated as having some concerns, mainly because of incomplete blinding and, in one study, participant attrition of about 21% (Marzouki et al., 2022; Mills et al., 2020). The three non-randomized controlled intervention studies were judged to have a moderate risk of bias (Caputo et al., 2018; Chu and Pan, 2012; Zanobini and Solari, 2019). (Bell 2021), whose comparison group consisted of typically developing children, was assessed separately under ROBINS-I (moderate risk of bias) but was not counted among the controlled intervention studies, because a non-equivalent developmental comparison characterizes differences between children with ASD and typically developing children rather than estimating the treatment effect of the aquatic intervention; this study therefore contributes within-group, qualitative information only. The eight single-group pre-post studies were considered to have a high risk of bias because of their uncontrolled design, absence of a comparator, and primary reliance on unblinded proxy-reported outcome measures; one of these studies (Alecu and Onea, 2025) used a non-validated author-developed questionnaire and was therefore excluded from all quantitative analyses. The three SCEDs were considered to be of generally acceptable methodological quality. Across the included studies, the most common methodological limitations were the lack of randomization and blinding, dependence on proxy-reported outcomes, and small sample sizes (Table 2, Figure 1). Tool-specific assessments are reported in Supplementary Table S2 (RoB 2, four RCTs) and Supplementary Table S3 (ROBINS-I and single-case criteria, 15 remaining studies); Figure 1 presents these judgments in separate panels by assessment framework; the panels use each tool's native domains and are not directly comparable across frameworks.

**Table 2:** Methodological quality of included studies.

References	Design	Appraisal tool	Overall judgment	Principal concerns
Albadry et al. (2026)	RCT (parallel)	RoB 2	Low	Therapist blinding not feasible
Pan (2010)	RCT (crossover)	RoB 2	Low	Teacher-rated outcomes; small sample
Marzouki et al. (2022)	RCT (parallel)	RoB 2	Some concerns	Limited detail on randomization; ~21% attrition
Mills et al. (2020)	RCT (crossover)	RoB 2	Some concerns	Small sample size; participants unblinded
Caputo et al. (2018)	Quasi-experimental	ROBINS-I	Moderate	Non-random allocation; confounding
Zanobini and Solari (2019)	Quasi-experimental	ROBINS-I	Moderate	Convenience sampling with matching
Chu and Pan (2012)	Non-randomized controlled	ROBINS-I	Moderate	Purposive allocation; non-blinded observers
Bell (2021)	Quasi-experimental (TD comparison)	ROBINS-I	Moderate	Typically developing comparison group; single session
Single-group pre-post (8)	Uncontrolled	ROBINS-I	High	No comparator; unblinded proxy reports; maturation effects
SCEDs (3)	Single-case	SCED RoB standards	Adequate	Limited replication in selected studies

RoB 2, Cochrane Risk-of-Bias tool 2.0; ROBINS-I, Risk Of Bias In Non-randomized Studies of Interventions; SCED RoB, single-case experimental design risk-of-bias standards; TD, typically developing. The eight single-group pre-post studies (including Alecu and Onea, 2025, excluded from pooling) and the three SCEDs are summarized together for brevity.

### 3.3 Study characteristics

The characteristics of the 19 included studies are summarized in Table 3. All studies were published between 2010 and 2026 and were conducted across 10 countries, with the largest proportion originating from the United States (eight studies). Collectively, the 18 publications reporting sample size described approximately 320 participants in total, with sample sizes ranging from 2 to 50 participants per study (median, 12). This figure represents the sum of publication-level sample sizes across 18 publications (one study did not report its sample size) and includes both participants with ASD and the typically developing comparison participants enrolled in one study (Bell, 2021); it may also overestimate the number of unique participants because two reports from the same research group (Alaniz et al., 2017, 2020) may have included overlapping samples. The number of unique participants with ASD is therefore uncertain. Regarding study design, four investigations were RCTs [two parallel-group (Albadry et al., 2026; Marzouki et al., 2022) and two crossover trials (Pan, 2010; Mills et al., 2020)], three were non-randomized controlled intervention studies (Caputo et al., 2018; Chu and Pan, 2012; Zanobini and Solari, 2019), one was a quasi-experimental study with a typically developing comparison group (Bell, 2021), eight were single-group pre-post studies or case series (Alaniz et al., 2017, 2020; Alecu and Onea, 2025; Ennis, 2011; Güeita-Rodríguez et al., 2021; Johnson et al., 2021; Oriel et al., 2016, 2020), and three employed SCEDs (Dowdy and Tincani, 2020; Pushkarenko et al., 2016; Vodakova et al., 2022). Participants ranged from preschool-aged to school-aged children, and Halliwick-based aquatic therapy was the most frequently applied intervention. Where reported, intervention sessions lasted between 30 and 90 min (most commonly 45–60 min), were delivered 0.5–3 times per week over periods ranging from 4 to 40 weeks, and were typically conducted in indoor heated pools with either a 1:1 therapist-to-child ratio or small-group instruction. Two reports from the same research group (Alaniz et al., 2017, 2020) may have included overlapping participants and are identified accordingly throughout the review.

**Table 3:** Characteristics of included studies (*N* = 19).

References	Country	Design	Intervention	*N*	Age, yr	Dose (min × /wk × wks)	ASD diagnostic basis
Alaniz et al. (2017)	USA	Single-group pre–post	Aquatic OT	7	6.1 (3–7)	60 × 1 × 24	Physician diagnosis or SSIS Autism Rating
Alaniz et al. (2020)	USA	Single-group pre–post	Aquatic OT	NR	3–7	≈ up to 24 sessions	NR
Albadry et al. (2026)	Egypt	RCT (parallel)	Aquatic training	50	4.0 (3–5)	45 × 3 × 12	CARS-2 (DSM-5 criteria)
Alecu and Onea (2025)	Romania	Single-group pre–post	Therapeutic swimming	36	11.3 (8–15)	45 × 2 × 8	DSM-5 + ADOS-2 (child psychiatrist)
Bell (2021)	USA	Quasi-experimental (TD comparison)	Aquatic psychosocial	32	9.7	Single session	CAST (screening tool)
Caputo et al. (2018)	Italy	Quasi-experimental	Multisystem aquatic (MAT)	26	≈8 (6–12)	45 × ≈1.3 × 40	DSM-5 criteria, ADI-R, ADOS
Chu and Pan (2012)	Taiwan	Non-randomized controlled	Halliwick/psychosocial	42	8.5 (7–12)	60 × — × 16	NR
Dowdy and Tincani (2020)	USA	SCED (A-B + FA)	Behavioral (pool transition)	2	13.5 (10–17)	NR	NR
Ennis (2011)	USA	Case series	Halliwick aquatic PT	6	(3–9)	60 × 1 × 10	NR
Güeita-Rodríguez et al. (2021)	Spain	Single-group pre–post	Halliwick (WST)	6	7.2 (6–12)	60 × 2 × ≈28	DSM-5
Johnson et al. (2021)	USA	Single-group pre–post	Aquatic psychosocial	10	(5.5–11)	30 × — × 3	NR
Marzouki et al. (2022)	Tunisia	RCT (parallel)	Aquatic training (Halliwick)	≈20	6.3 (6–7)	— × 2 × 8	DSM-V criteria + CARS (score > 30)
Mills et al. (2020)	Australia	RCT (crossover)	Hydrotherapy	8	8.7 (6.8–12.6)	45 × 1 × 4	Medical doctor or specialist diagnosis
Oriel et al. (2016)	USA	Single-group pre–post	Aquatic exercise	11	6.6 (3–11)	60 × 2 × 4	DSM-IV (not further specified)
Oriel et al. (2020)	USA	Single-group pre–post	Aquatic exercise/social	13	7.7 (7–10)	60 × 1 × 8	Clinical diagnosis (instrument NR)
Pan (2010)	Taiwan	RCT (crossover)	Halliwick/psychosocial	16	7.2 (6–9)	90 × — × 10	NR
Pushkarenko et al. (2016)	Canada	SCED (A-B-A)	Aquatic psychosocial	3	(11–16)	30–40 × — × 13	NR
Vodakova et al. (2022)	Czech Rep.	SCED (multiple baseline)	Halliwick	7	9.4 (7–12)	60 × 1 × 7	ICD-10 (neuropsychiatrist)
Zanobini and Solari (2019)	Italy	Quasi-experimental (matched)	Swimming (Halliwick)	25	5.6 (3–8)	30 × 0.5 × ≈20	ICD-10/DSM-IV/DSM-5/DC:0-3, confirmed by ABC ≥ 53

OT, occupational therapy; PT, physiotherapy; MAT, multisystem aquatic therapy; WST, water-specific therapy; FA, functional analysis; SCED, single-case experimental design; NR, not reported; —, not reported/not applicable. Age is presented as mean (range) where available. Dose = minutes per session × sessions per week × total weeks. The two crossover studies (Mills et al., 2020; Pan, 2010) were included among the four RCTs. Values preceded by ≈ were estimated or derived from incomplete reporting; the summed total (approximately 320 participants, including typically developing comparison participants where enrolled) excludes the one study Alaniz et al., (2020) that did not report its sample size; the number of unique participants with ASD is uncertain. Alaniz et al. (2017) and Alaniz et al. (2020) may include overlapping participants.

### 3.4 Types of intervention

Nearly all interventions were based on, or incorporated components of, the Halliwick method; other approaches included multisystem aquatic therapy (Caputo et al., 2018), therapeutic swimming (Alecu and Onea, 2025), aquatic occupational therapy (Alaniz et al., 2017, 2020), and water-specific therapy (Güeita-Rodríguez et al., 2021). The general structure of the programs was broadly similar: sessions lasted 30–90 min, were delivered once to three times per week, continued for 4–40 weeks, and were paced according to the individual child. Several programs had a psychosocial orientation and included explicit opportunities for social engagement; one intervention used trained peers and siblings as in-water partners (Chu and Pan, 2012), and two were conducted as crossover trials in which each group alternated between aquatic intervention and usual activities (Pan, 2010; Mills et al., 2020). Two SCEDs directly targeted challenging behavior through behavioral protocol in the aquatic setting (Dowdy and Tincani, 2020; Pushkarenko et al., 2016).

### 3.5 Measures

The 19 studies applied more than 15 different outcome instruments, and no single measure was used in a directly poolable form across three or more studies. Behavioral and autistic-symptom severity was assessed using the Childhood Autism Rating Scale (CARS/CARS-2; Albadry et al., 2026; Caputo et al., 2018), the Gilliam Autism Rating Scale (GARS-2; Marzouki et al., 2022), the Aberrant Behavior Checklist (Zanobini and Solari, 2019), the School Social Behavior Scales (SSBS-2; Pan, 2010), and the PDD Behavior Inventory (PDDBI; Oriel et al., 2016). Social-emotional functioning was measured using the Vineland Adaptive Behavior Scales (Caputo et al., 2018), the Home and Community Social Behavior Scales (Oriel et al., 2020), the Pictorial Scale of Perceived Competence (Güeita-Rodríguez et al., 2021), and the Social Responsiveness Scale (Zanobini and Solari, 2019). Emotion-related functioning was evaluated using the Child Behavior Checklist (Mills et al., 2020), the Emotion Regulation Checklist (Marzouki et al., 2022), and PDDBI arousal subscales (Oriel et al., 2016), whereas quality of life was assessed with the Pediatric Quality of Life Inventory (Albadry et al., 2026; Ennis, 2011; Güeita-Rodríguez et al., 2021) and a general-health index (Johnson et al., 2021). Where reported, the controlled studies described acceptable validity and reliability for their instruments; two single-group studies did not provide these properties.

### 3.6 Outcome

Across all four outcome domains, the direction of effect was predominantly favorable toward aquatic intervention; a study-by-domain summary is presented in Table 4 and illustrated as an effect-direction (harvest) plot in Figure 3. Because the outcome measures were heterogeneous and only a small number of controlled studies were available, no meta-analysis was conducted; the findings are therefore synthesized narratively, with the direction and statistical significance of effects tabulated for each study. Study-level standardized effect sizes with 95% confidence intervals for the controlled comparisons are shown, without a pooled estimate, in Supplementary Figure S1; their dispersion within the behavioral domain (*d* = 0.63 to *d* = 3.87) illustrates why a single summary estimate would be misleading. The domains differed substantially, however, in the amount of controlled evidence supporting them, and this distinction is described below. Throughout this review, the term “controlled study” denotes a design in which the aquatic intervention is compared with a control condition in order to estimate a treatment effect; a comparison with typically developing children does not meet this definition, because it characterizes developmental differences rather than estimating the effect of the intervention. Bell (2021), the only included study relying solely on such a comparison, was therefore not counted among the controlled intervention studies in any domain and is reported qualitatively only.

**Table 4:** Summary of the findings: direction of effect by study and outcome domain.

Study	D1 Emotion	D2 Social-emotional	D3 Behavioral	D4 Quality of life
Alaniz et al. (2017)		+	0	
Alaniz et al. (2020)		0		
Albadry et al. (2026)			+^*^	+^*^
Alecu and Onea (2025)°	+^*^°	+ °	+ °	+^*^°
Bell (2021)†	0	+^*^		
Caputo et al. (2018)		+	+^*^	
Chu and Pan (2012)		+^*^		
Dowdy and Tincani (2020)			+^*^	
Ennis (2011)	+	+		+
Güeita-Rodríguez et al. (2021)		+^*^		+
Johnson et al. (2021)	+	0	+	0
Marzouki et al. (2022)	+		+^*^	
Mills et al. (2020)	+^*^		+	
Oriel et al. (2016)	+^*^	+	+^*^	
Oriel et al. (2020)		+^*^	0	
Pan (2010)	+^*^	+	+^*^	
Pushkarenko et al. (2016)			+^*^	
Vodakova et al. (2022)			+	
Zanobini and Solari (2019)		+^*^	+^*^	

+^*^ = statistically significant favorable change; + = favorable change without statistical significance; 0 = no change or mixed findings; blank = domain not assessed. °Alecu and Onea (2025) used a non-validated, author-developed instrument and was excluded from all quantitative analyses: it contributes to no effect-direction count, no GRADE judgment, and no entry in the forest display. ‡Bell (2021) used a typically developing comparison group and therefore does not estimate a between-group treatment effect of aquatic intervention; its entries reflect within-group change only, and the study is not counted among the controlled intervention studies in any domain or GRADE judgment. Alaniz et al. (2017) and Alaniz et al. (2020) may include overlapping participants and should therefore be considered as one body of evidence. Effect sizes were oriented so that positive values indicated improvement, regardless of the original scale direction.

**Figure 3:**
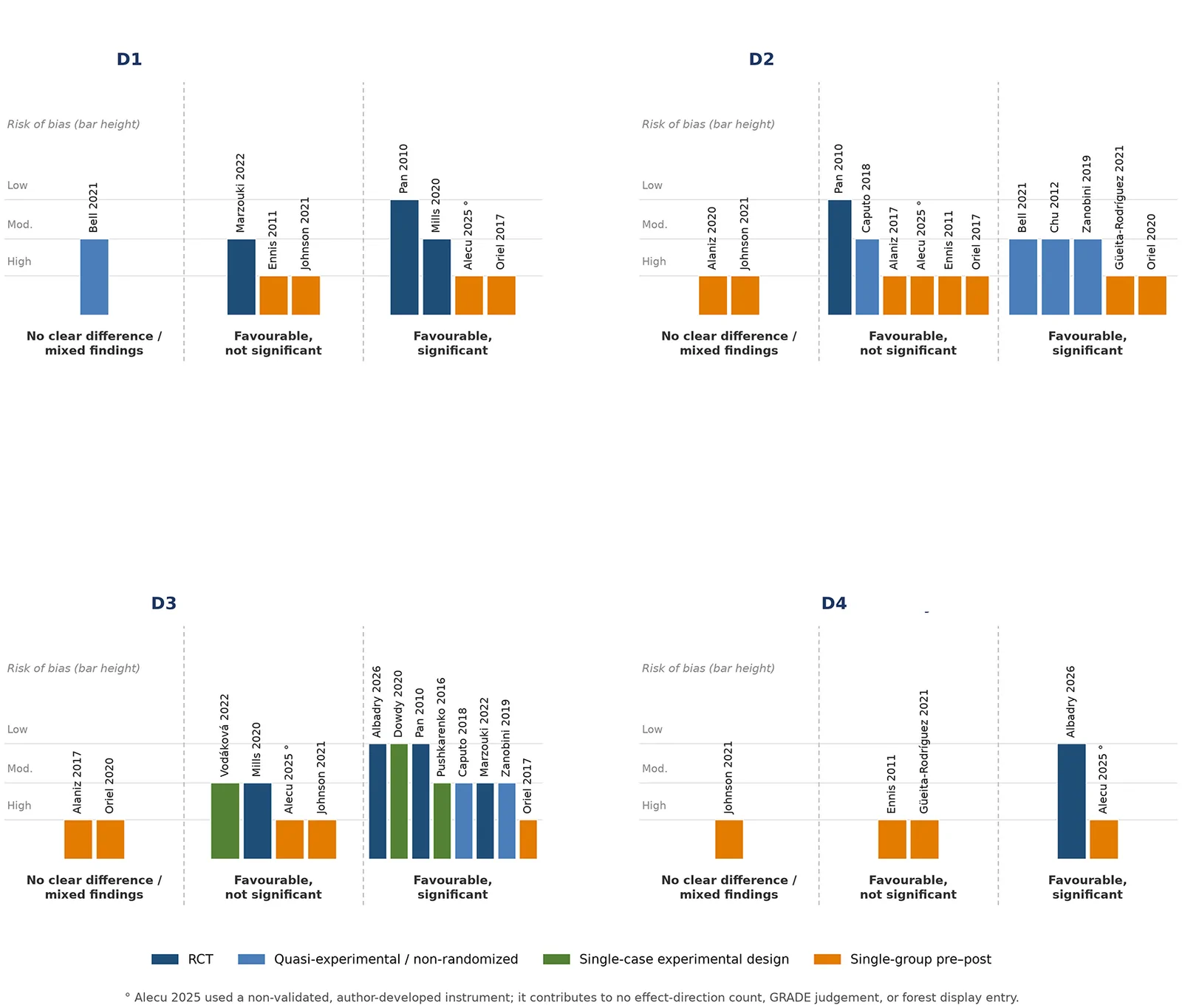
Effect-direction (harvest) plot of the direction of effect by study and outcome domain. Bar height denotes overall risk of bias (taller = lower risk); color denotes study design; horizontal position denotes the direction and significance of effect. **(D1)** Emotion-related functioning. **(D2)** Social-emotion functioning. **(D3)** Behavioral outcomes. **(D4)** Quality of life. No study favored the control condition. However, the almost universally favorable direction should be interpreted with caution because most studies had a high risk of bias (orange), and outcome measures were highly fragmented.

### Domain 1—emotion-related functioning

First, seven studies reported emotion-related outcomes, and all showed findings favoring aquatic intervention; however, because the limited controlled studies assessed different constructs with different instruments, no meta-analysis was conducted for this domain. Among the strongest controlled findings, the crossover trial by Mills and colleagues reported decreases in parent-rated internalizing and total problems on the CBCL (Mills et al., 2020), while Pan found a large reduction in teacher-rated hostile or irritable behavior (Pan, 2010). Among uncontrolled studies, Oriel and colleagues reported significant pre-post improvements in arousal-regulation problems and specific fears on the PDDBI (Oriel et al., 2016), and Johnson and colleagues observed small decreases in anxiety-related subscales (Johnson et al., 2021). The only controlled trial that directly measured cognitive reappraisal using the Emotion Regulation Checklist found a between-group effect close to zero (Marzouki et al., 2022), although the study was underpowered.

### Domain 2—social-emotional functioning

Second, 11 studies examined social-emotional functioning. Significant favorable effects were reported for eye contact, phrase use, and smiling (Bell, 2021), for substantially greater peer- and teacher-directed interaction under peer-assisted conditions (Chu and Pan, 2012), for social competence and peer relationships (Oriel et al., 2020), for perceived competence (Güeita-Rodríguez et al., 2021), and—most importantly for a controlled comparison—for an ABC social-relating time-by-group interaction favoring the swimming group (Zanobini and Solari, 2019). Four of these studies contributed controlled between-group comparisons; three provided extractable data on standardized social instruments (Pan, 2010; Caputo et al., 2018; Zanobini and Solari, 2019), and the fourth reported peer- and teacher-directed interaction under peer-assisted conditions (Chu and Pan, 2012); because these comparisons used different social instruments (the Vineland, ABC, and SSBS-2 social scales), they were not combined quantitatively. Across all 11 studies, the direction of effect was favorable, but in most studies it did not reach statistical significance; thus, the social-emotional evidence is based on a consistent favorable direction rather than a measurable common effect.

### Domain 3—behavioral outcomes

Third, the behavioral domain contained the largest evidence base (13 studies) and showed the most consistent favorable direction. Among controlled studies, significant favorable effects were found for reduced autism severity on the CARS-2 (Albadry et al., 2026), reduced CARS severity accompanied by gains in adaptive behavior (Caputo et al., 2018), reduced GARS-2 stereotypy (Marzouki et al., 2022), decreased teacher-rated antisocial behavior on the SSBS-2 (Pan, 2010), and lower ABC total and SRS mannerism scores (Zanobini and Solari, 2019). Uncontrolled and single-case studies showed the same general direction, with the largest single-study improvements reported on the PDDBI (Oriel et al., 2016), and functional reductions in challenging behavior observed in both behavior-targeted SCEDs (Dowdy and Tincani, 2020; Pushkarenko et al., 2016). All six controlled studies that provided a between-group behavioral comparison favored aquatic intervention; five reached statistical significance, whereas the remaining trial—a crossover RCT (Mills et al., 2020)—favored aquatic intervention but was not statistically significant (Figure 3, Table 4). These instruments measure related but non-identical constructs (severity, stereotypy, and problem behavior), and the studies differed considerably in design and intervention dose; therefore, the favorable signal reflects a consistent direction across heterogeneous measures rather than a measurable common effect, and was not pooled. Importantly, only one of the six controlled studies used an active land-based comparator (Albadry et al., 2026), meaning that the favorable behavioral signal mainly reflects aquatic intervention vs. little or no intervention rather than a water-specific effect. Taken together, the behavioral evidence indicates a consistently favorable direction across studies but, because effects could not be pooled, is based on vote-counting and should be considered hypothesis-generating.

### Domain 4—quality of life and wellbeing

Finally, five studies examined quality-of-life outcomes. The only controlled estimate reported a significant improvement in the parent-proxy PedsQL total score favoring aquatic training (Albadry et al., 2026); because only one controlled study was available, no pooled analysis was conducted. Among uncontrolled studies, one reported non-significant improvements in PedsQL total and subscale scores (Güeita-Rodríguez et al., 2021), a case series described PedsQL improvement in four of six children (Ennis, 2011), and another study found minimal change in a parent-rated general-health index (Johnson et al., 2021). Overall, the evidence for this domain is suggestive but limited.

### 3.7 Additional outcomes

Adverse events. No serious adverse events were reported in any included study; the reported events were isolated and minor, such as transient gastrointestinal discomfort after swallowing pool water and one episode of aggression that led to withdrawal (Oriel et al., 2016). This finding should be interpreted with caution, however, because most studies did not formally monitor or pre-define adverse events; therefore, the apparent safety reflects limited reporting as much as demonstrated safety. ^**^Adherence and dropout.^**^ Where reported, attendance was high [100% in two studies (Albadry et al., 2026; Güeita-Rodríguez et al., 2021), and 92–94% in others (Alecu and Onea, 2025; Oriel et al., 2020)], and dropout was generally low [no dropouts in several controlled studies; 5.7% in one RCT (Albadry et al., 2026)], although higher attrition was observed in two uncontrolled studies [21.4% and 45.5% (Ennis, 2011; Oriel et al., 2016)], usually because of scheduling difficulties or unrelated medical reasons. ^**^Satisfaction.^**^ Caregiver-reported satisfaction and perceived benefit were consistently positive in studies that measured these outcomes (Mills et al., 2020; Güeita-Rodríguez et al., 2021; Oriel et al., 2020)

### 3.8 Consistency of effect direction, risk of bias, and reporting bias

The four outcome domains varied in the amount of controlled evidence supporting them and in the consistency of the favorable direction. The behavioral and social-emotional domains had the richest evidence base, whereas the emotion-related and quality-of-life domains relied on only one or two controlled studies. Across the review, the direction of effect was favorable in most studies, and no study favored the control condition (Figure 3, Table 4); however, this almost universal favorable pattern should be interpreted cautiously because the evidence base was dominated by studies with a high risk of bias (eight of nineteen) or without a concurrent comparator (Figure 1), and such uniformity may itself indicate possible reporting or publication bias. When the analysis was limited to randomized and non-randomized controlled studies, the favorable direction remained, but fewer comparisons achieved statistical significance, suggesting that the strongest impressions of benefit were derived disproportionately from weaker study designs. Because no effects were pooled, formal small-study assessments (funnel plots and Egger's test) were not applicable; the largest reported effects were observed in studies using non-validated, author-constructed instruments, further supporting the concern that the apparent magnitude of benefit may be inflated in the lower-quality literature.

### 3.9 Certainty of the evidence

Using GRADE, the certainty of evidence was rated as low to very low across all four domains (Table 5); the full evidence profile—contributing outcomes, number and design of studies, participant numbers, comparator types, the judgment and reason for each of the five GRADE domains, and the resulting certainty rating—is provided in Supplementary Table S1. Downgrading decisions were made independently for each GRADE domain, and no domain was permitted to compensate for another. Each domain was rated starting from high certainty, because randomized evidence contributed to every domain; the presence of non-randomized designs was accounted for within the risk-of-bias domain rather than by lowering the starting rating. Risk of bias was rated serious in all four domains, reflecting the predominance of uncontrolled designs, lack of blinding, and reliance on proxy-reported outcomes. Imprecision was assessed from the number of contributing participants and the width of the study-level 95% confidence intervals (Supplementary Figure S1), specifically whether the plausible range of effects included both little or no benefit and potentially important benefit; the inability to pool was not itself treated as grounds for downgrading. On this basis, imprecision was rated serious in the behavioral, social-emotional, and emotion-related domains (small total samples with wide intervals spanning negligible to important benefit) and very serious in the quality-of-life domain, for which a single small controlled study was available. Inconsistency was not rated down in the behavioral domain, where all six controlled comparisons were concordant in direction; this concordance was treated as relevant only to the inconsistency domain and was not used to offset downgrading for risk of bias or imprecision. Indirectness related to heterogeneous instruments measuring related but non-identical constructs contributed to downgrading in the emotion-related domain. Publication bias was suspected on descriptive grounds—the near-universal favorable direction and the concentration of the largest effects in studies using non-validated instruments—but, in the absence of a pooled analysis, was not formally rated down in any domain. The behavioral domain, supported by the largest body of controlled evidence, was accordingly rated low certainty (downgraded once for risk of bias and once for imprecision). In line with guidance on synthesis without meta-analysis and vote-counting by direction of effect (Campbell et al., 2020; McKenzie and Brennan, 2024), the resulting certainty ratings should be regarded as approximate.

**Table 5:** Body-of-evidence and GRADE certainty summary, by outcome domain.

Domain	Evidence base (controlled/total)	Direction of effect (studies favorable/reporting)	Certainty	Principal reasons for downgrading
D1 Emotion	3/7 (1 parallel RCT, 2 crossover RCTs)	All 3 controlled comparisons favorable, 2 statistically significant (not pooled)	Very low (⊕○○○)	RoB (−1); indirectness: heterogeneous constructs and instruments (−1); imprecision: very few small controlled studies with wide CIs spanning negligible to important benefit (−1)
D2 Social-emotional	4/11 (1 crossover RCT, 3 non-randomized/quasi)	All 4 controlled comparisons favorable, 2 statistically significant (not pooled)	Low (⊕⊕○○)	RoB (−1); imprecision: few small studies with wide CIs spanning negligible to important benefit (−1)
D3 Behavioral	6/13 (2 parallel RCTs, 2 crossover RCTs, 2 quasi-experimental)	All 6 controlled comparisons favorable, 5 statistically significant (not pooled)	Low (⊕⊕○○)	RoB (−1); imprecision: small total sample, wide CIs spanning negligible to important benefit (−1); no downgrade for inconsistency (direction concordant); reporting bias suspected but not rated down
D4 Quality of life	1/5 (1 parallel RCT)	Single controlled study favorable and statistically significant (not pooled)	Very low (⊕○○○)	RoB (−1); very serious imprecision: single small controlled study (−2)

RoB, risk of bias; CI, confidence interval. No quantitative pooling was performed; the direction of effect was synthesized through vote-counting (see Table 3 and Figure 3). Controlled counts denote intervention-effect comparisons; Bell (2021) (typically developing comparison group) is excluded from all controlled counts and GRADE judgments and contributes qualitative within-group information only. Certainty ratings follow from the stated downgrading levels: each domain was rated starting from high certainty and then downgraded as shown; ⊕⊕○○ low; ⊕○○○ very low. Imprecision was judged from the number of contributing participants and the width of the study-level 95% CIs (Supplementary Figure S1)—specifically, whether plausible effects spanned negligible to potentially important benefit—and not from the inability to perform a meta-analysis. Directional concordance informed only the inconsistency judgment and was not used to offset downgrading for risk of bias, imprecision, or publication bias.

## 4 Discussion

In this systematic review, a structured narrative synthesis without meta-analysis was applied to 19 included studies to assess the effects of aquatic interventions on emotional and behavioral outcomes in children with ASD. To our knowledge, this is the first prospectively registered systematic review to examine aquatic interventions across four distinct emotion- and behavior-related domains in children with ASD and to emphasize the distinction between a water-specific effect and the broader effect of structured activity; previous reviews considered hydrotherapy for social and behavioral aspects (Mortimer et al., 2014), or motor and aquatic-skill outcomes (van t Hooft et al., 2024; Aleksandrovic et al., 2016), without separating these questions. A meta-analysis was not performed because the outcome instruments were too fragmented and the number of controlled studies was too small to provide a comparable pooled estimand; therefore, the direction of effect was tabulated for each study. Two findings were particularly notable. First, the direction of effect was favorable in every domain—based on vote-counting rather than pooled estimates, and no study favored the comparator, representing an unusually consistent signal for an intervention that also appeared feasible and acceptable in the included studies, with generally high reported attendance and low dropout, although harms were not systematically monitored. Second, this favorable direction followed a clear gradient of evidentiary strength: the evidence was densest and most internally consistent for behavioral outcomes (all six controlled studies favorable in direction, five reaching significance), favorable but non-significant for social-emotional functioning, and too sparse to pool for emotion-related functioning and quality of life. The appropriate conclusion is therefore to specify which outcomes the current evidence can support, and with what degree of confidence, rather than to make a broad claim of effectiveness; precision was deliberately not created by pooling uncontrolled single-arm data. The main findings are discussed below.

### 4.1 Effect on behavioral outcomes

First, behavioral outcomes provided the largest body of evidence and the most consistent favorable direction; all six controlled studies favored aquatic intervention in direction, and five showed statistically significant effects. This finding is consistent with the broader physical-activity literature in ASD, in which Healy and colleagues reported an overall moderate pooled effect across outcomes (*g* = 0.62; Healy et al., 2018), and Wang and colleagues observed a moderate pooled effect specifically on repetitive behaviors (ES = 0.553; Wang et al., 2023). Because behavioral outcomes were assessed using heterogeneous instruments, they were not pooled; the favorable signal therefore depends on the consistent direction of effect across studies and should be interpreted as hypothesis-generating rather than as a reliable effect size. If a benefit is present, three hypothesized mechanisms merit investigation: hydrostatic pressure and mildly warm water temperature have been proposed to reduce sympathetic tone through parasympathetic activation (Mooventhan and Nivethitha, 2014; Haghayegh et al., 2019), which might in turn attenuate the sensory-overload-driven reinforcement of stereotypic behavior; rhythmic aquatic movement may provide structured vestibular and proprioceptive input that has been hypothesized to support basal-ganglia-cortical regulation of motor execution (Gandhi and Lee, 2021); and the inherent structure of the aquatic setting may create a predictable sensory-cognitive environment that could reduce behavioral stress reactivity. A major caveat is that only one included trial used an active land-based comparator (Albadry et al., 2026), and that study was the statistical outlier; consequently, the data cannot establish whether the observed effect reflects a water-specific mechanism or the general effect of structured exercise. Another consideration is that two of the six controlled trials (Mills and Pan) used crossover designs; for interventions targeting skills and behavioral regulation, gains acquired during the aquatic phase may not wash out before the comparison phase, so carryover and practice effects could inflate the apparent between-condition difference. Therefore, the signals from these two trials—one of which contributed several stronger behavioral effects—were weighted cautiously.

### 4.2 Effect on social-emotional functioning

Second, the social-emotional signal was favorable in direction but could not be quantified; this pattern is broadly consistent with the moderate benefits for social functioning reported in physical-activity meta-analyses in ASD (Healy et al., 2018). The water environment may offer a distinctive social scaffold: buoyancy may reduce gravitational load so that children begin from a relatively equalized motor baseline, which could lessen the social withdrawal that motor-coordination difficulties can provoke on land; water temperature and tactile feedback may lower physiological arousal in social settings; and structured aquatic games, such as the Halliwick ten-point program, provide clear social roles and turn-taking rules. Consistent with previous observations, improvements may be partly driven by the enforced, instructor-guided social interaction inherent in group aquatic programs (Chu and Pan, 2012). Future studies should use validated standardized measures rather than non-validated instruments to quantify these social-emotional effects.

### 4.3 Effect on emotion-related functioning and quality of life

Third, although the direction was favorable, neither the emotion-related nor the quality-of-life domain could be meta-analyzed, as each included only one or two controlled studies using heterogeneous instruments. Mechanistically, aquatic intervention may mainly improve arousal-related emotional symptoms through bottom-up parasympathetic activation, while having a more limited effect on emotion-regulation capacities that depend on top-down cognitive reappraisal; consistent with this interpretation, the only controlled trial that directly measured cognitive reappraisal reported a near-zero between-group effect (Marzouki et al., 2022), although it was underpowered. These interpretations are speculative in the absence of mechanistic data. Future studies should use unified, validated composite measures so that these domains can become suitable for quantitative synthesis.

### 4.4 Mechanisms and the water-vs.-land question

Finally, the results do not confirm the central prediction of the sensory-regulation account. This account predicts that the largest and most consistent effects should appear in the most proximal domain, emotion-related functioning; instead, the densest and most directionally consistent evidence was found in the behavioral domain, whereas the emotion domain was too sparse to support any firm conclusion. This ordering should nevertheless be interpreted carefully, because it is confounded by uneven study availability: the behavioral domain simply contained more studies and more controlled evidence, so its apparent prominence may reflect research focus rather than a true mechanistic gradient. The observed pattern is therefore better treated as a hypothesis for future investigation than as confirmation of the framework. Whether water provides benefits beyond those of structured exercise remains the central unresolved question and can be answered only by trials that directly compare aquatic intervention with an active land-based control while also measuring the proposed autonomic mediators.

## 5 Limitations

Several limitations should be considered. First, the evidence base is dominated by uncontrolled designs with a high risk of bias. Second, because the outcome instruments were highly fragmented—none was used in a directly comparable form across three or more studies—and controlled studies were limited, a meta-analysis was not performed; the synthesis therefore depended on the direction of effect (vote-counting), which ignores the magnitude and precision of effects, gives equal weight to a small high-quality trial and a small uncontrolled series, and remains vulnerable to reporting bias. Third, just over one quarter of the reports sought for full-text assessment (35 of 134) could not be retrieved despite institutional searches, interlibrary-loan requests, and direct correspondence with authors (Supplementary Table S4); availability bias therefore cannot be excluded, and the direction of the missing evidence is unknown. Fourth, only one included trial used an active land-based comparator, so the observed associations cannot be attributed specifically to the water medium rather than to structured activity, attention, or expectation effects. A further consideration is interpretive rather than procedural: much of the heterogeneity that prevented pooling reflects the clinical reality of autism spectrum disorder—a markedly heterogeneous condition for which individualized, rather than standardized, intervention is the norm. Viewed in this light, the consistency of effect direction observed across otherwise dissimilar samples, instruments, and settings constitutes a potentially informative, hypothesis-generating pattern rather than evidence more robust than a pooled estimate; it remains vulnerable to selective outcome reporting, publication bias, small-study effects, and the equal weighting of studies of markedly different methodological quality, and the underlying evidence remains of low to very low certainty for the reasons set out above. Given these constraints, the findings should be applied cautiously.

## 6 Conclusion

### 6.1 Implications for clinical practice

An emerging but low-certainty body of evidence suggests that structured aquatic interventions, often based on the Halliwick approach, are associated with favorable changes across emotional, social-emotional, behavioral, and quality-of-life domains in children with ASD, with the most consistent direction of effect observed in the behavioral domain. Because nearly all controlled studies compared aquatic intervention with no intervention or usual care rather than with an active alternative, any benefit should be interpreted as relative to little or no intervention and not as evidence of a water-specific effect. The generally high attendance and absence of reported serious adverse events suggest preliminary feasibility and acceptability of aquatic interventions as an adjunct option; however, because most studies did not formally monitor or prespecify harms, safety remains insufficiently characterized. Because the evidence base is small, dominated by uncontrolled designs, limited by fragmented outcome measures that could not be pooled, and based on vote-counting rather than quantified effects, clinical recommendations should be made with caution, and aquatic intervention should be regarded as a promising adjunct rather than an established treatment. Implementation also depends on access considerations, including local service models and funding.

### 6.2 Implications for future research

The priorities for the field are clear. Adequately powered, multicenter randomized controlled trials are required, incorporating an active land-based comparator to isolate any water-specific effect and including objective physiological measures such as heart-rate variability to test the proposed sensory-regulation mechanism. These trials should also be designed and reported in a way that allows the pre-specified moderators of effect—intervention type, intervention duration, participant age, and ASD severity—to be formally examined, which the current evidence base was too sparse to support. The development and adoption of an international core outcome set would address the instrument fragmentation that currently prevents meta-analysis in two of the four domains and would discourage the use of non-validated, author-constructed instruments. Future studies should also report ASD severity consistently, include longer-term follow-up to assess the durability of effects, and apply current diagnostic criteria in participant sampling. Until such evidence becomes available, conclusions about the effects of aquatic interventions for children with ASD should remain provisional. By mapping the strength of evidence domain by domain and specifying the active-comparator trials, physiological mediators, and core outcome set needed to close current gaps, this review provides a roadmap for moving aquatic-intervention research from promising but low-certainty signals toward controlled, mechanistically informative evidence.

## Data Availability

The original contributions presented in the study are included in the article/Supplementary material, further inquiries can be directed to the corresponding author.
