# Supplementary material for: The effects of aquatic interventions on emotional and behavioral outcomes in children with autism spectrum disorder
Source: Front Psychol. 2026 Sep 23;17:1929354. doi: 10.3389/fpsyg.2026.1929354 (PMC13642286; doi:10.3389/fpsyg.2026.1929354)
Supplement: Supplementary file 1 [file Data_Sheet_1.pdf]

Supplementary Materials

Figure S1. Forest-style display of study-level effect sizes by outcome domain

No pooled estimate was computed due to heterogeneity. Effect sizes are standardized mean differences (Cohen's d; see Methods S6). ° = uncontrolled pre–post (within-group change only). 95% CIs estimated from sample sizes where not reported. Bell 2021 (developmental comparison, ASD vs. typically developing) is not included.

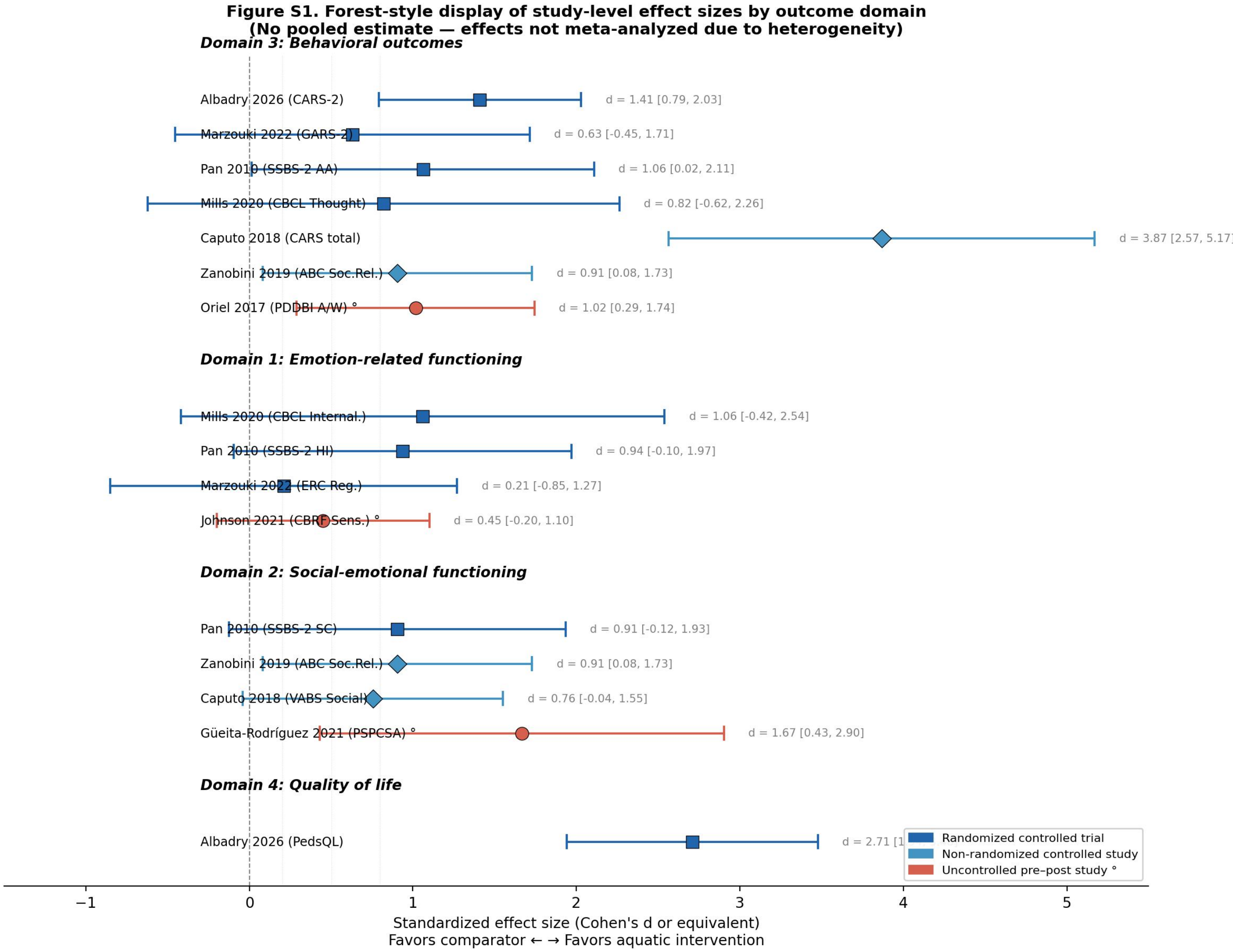

° Uncontrolled studies: effect sizes reflect within-group pre–post change only (no between-group comparison).
Effect sizes converted to Cohen's d equivalents: from η^2p via $d = 2\sqrt{\eta^2p/(1-\eta^2p)}$; from r via $d = 2r/\sqrt{1-r^2}$.
95% CIs estimated from sample sizes where not reported by the original studies.

Table S1. GRADE Summary of Findings (evidence profile)

⊕ ⊕ ⊕ ⊕ High; ⊕ ⊕ ⊕○ Moderate; ⊕ ⊕○○ Low; ⊕○○○ Very low. Certainty ratings follow from the stated downgrading levels: each domain was rated starting from high certainty, because randomized evidence contributed to every domain, and the presence of non-randomized designs was accounted for within the risk-of-bias domain rather than by lowering the starting rating. Imprecision was judged from the number of contributing participants and the width of the study-level 95% confidence intervals (Figure S1)—specifically, whether plausible effects spanned negligible to potentially important benefit—and not from the inability to perform a meta-analysis. Directional concordance informed only the inconsistency judgement and was not used to offset downgrading for risk of bias, imprecision, or publication bias. a Study-level standardized effect sizes (Cohen's d) for the six controlled behavioral comparisons ranged from 0.63 to 3.87 (Figure S1); this dispersion, together with heterogeneity of design, comparator and instrument, precludes a meaningful summary estimate. b A uniformly favorable direction across small studies with a high risk of bias may indicate reporting or publication bias; this concern was considered descriptively but, in the absence of a pooled analysis, was not formally rated down. c Bell 2021 used a typically developing comparison group, which does not estimate a treatment effect of aquatic intervention; it is reported for transparency, is excluded from all controlled counts and GRADE judgements, and contributes qualitative within-group information only.

**Outcome domain**	**Studies (design)**	**N**	**Comparator**	**Direction of effect**	**Risk of bias**	**Inconsistency**	**Indirectness**	**Imprecision**	**Pub. bias**	**Certainty**
**D1: Emotion-related**	7 (3 controlled RCTs: 1 parallel, 2 crossover; 3 uncontrolled; 1 developmental comparison c)	~100	No intervention/TAU	Favorable 6/7; controlled: all 3 favorable, 2 sig.	Serious (−1): most evidence uncontrolled; proxy-reported outcomes	Not serious	Serious (−1): heterogeneous constructs and instruments	Serious (−1): only 3 small controlled studies; wide CIs spanning negligible to important benefit a	Suspected descriptively; not rated down b	⊕○○○ **VERY LOW**
**D2: Social-emotional**	11 (4 controlled: 1 crossover RCT, 3 NR/quasi; 6 uncontrolled; 1 developmental comparison c)	~220	No intervention/TAU; attention control	Favorable 9/11 (excl. Bell 2021); controlled: all 4 favorable, 2 sig.	Serious (−1): few blinded; proxy-reported outcomes	Not serious	Not serious	Serious (−1): few small studies; wide CIs spanning negligible to important benefit a	Suspected descriptively; not rated down b	⊕ ⊕○○ **LOW**
**D3: Behavioral**	13 (6 controlled: 4 RCTs [2 parallel, 2 crossover], 2 quasi-experimental; 4 uncontrolled; 3 SCEDs)	~280	No intervention/TAU (1 active land)	All 6 controlled favorable; 5/6 sig.	Serious (−1): only 2 low-risk RCTs	Not serious: direction concordant across all 6 controlled comparisons	Not serious	Serious (−1): small total sample, wide CIs spanning negligible to important benefit a	Suspected descriptively; not rated down b	⊕ ⊕○○ **LOW**
**D4: Quality of life**	5 (1 parallel RCT; 4 uncontrolled/case series)	~80	Active land (1 RCT); none (4)	Favorable 4/5; single controlled study favorable and sig.	Serious (−1): only 1 controlled study	Not serious	Not serious	Very serious (−2): single small controlled study a	Suspected descriptively; not rated down b	⊕○○○ **VERY LOW**

Table S2. Risk of bias assessment for randomized controlled trials (Cochrane RoB 2)

Each cell contains the judgment and the supporting rationale. Green = low risk; yellow = some concerns; red = high risk. Assessments are outcome-specific where relevant.

Study — Outcome	D1: Randomization process	D2: Deviations from intended interventions	D3: Missing outcome data	D4: Measurement of the outcome	D5: Selection of the reported result	Overall
Albadry 2026 — CARS-2 / PedsQL	Low risk: Computerized randomization (GraphPad); allocation concealed by independent researcher	Some concerns: Therapist blinding not feasible due to intervention nature; no protocol deviations reported	Low risk: 3/53 (5.7%) dropouts; low and balanced between groups	Low risk: Outcome assessors blinded to group allocation throughout study; standardized procedures	Low risk: All pre-specified outcomes reported with complete data	Low risk
Pan 2010 — SSBS-2	Low risk: Adequate sequence generation; participants divided into groups of equal size and disability type; allocation concealed	Low risk: Participants unblinded but no deviations from the intended intervention; carryover risk inherent in the crossover design is addressed separately in the Discussion	Low risk: All 16 participants completed the 21-week program; no missing data	Low risk: SSBS-2 rated by classroom teachers blinded to treatment allocation	Low risk: All social and antisocial behavior subscales reported	Low risk: adequate sequence generation, assessor blinding to allocation, and complete follow-up
Marzouki 2022 — GARS-2	Some concerns: Stated 'randomly divided' only; no sequence generation details; no allocation concealment reported	Some concerns: Therapist blinding not feasible; control group told not to do aquatic activities — expectation effects possible	Some concerns: 6/28 (21.4%) excluded for health reasons; exceeds 10% threshold, though stated unrelated to intervention	Some concerns: GARS-2 parent-reported; parents aware of group allocation; no assessor blinding documented	Low risk: All 3 pre-specified outcomes (TGMD-2, GARS-2, ERC) fully reported	Some concerns: D1, D3, D4 all some concerns
Marzouki 2022 — ERC	Some concerns: As above	Some concerns: As above	Some concerns: As above	Some concerns: ERC parent-reported; parents aware of allocation	Low risk: As above	Some concerns
Mills 2020 — CBCL	Low risk: Microsoft Excel randomization function; groups balanced at baseline	Some concerns: Participants not blinded to aims or allocation; crossover — possible carryover for behavioral regulation outcomes	Low risk: All 8 participants completed; no missing data; 100% attendance with make-up sessions	Some concerns: CBCL parent-reported; parents aware of treatment condition. However, researchers blinded to CBCL results via sealed envelopes	Low risk: All CBCL syndrome scales and domain summaries reported	Some concerns: D2 and D4 some concerns

Table S3. Risk of bias assessment for non-randomized and uncontrolled studies

Controlled non-randomized studies: ROBINS-I (Sterne et al., 2016). Single-group studies: adapted ROBINS-I framework. SCEDs: design-specific standards (WWC, 2020). Each cell contains the judgment and supporting rationale. Tools are not directly interchangeable across designs. ° Alecu 2025 used non-validated instrument; excluded from quantitative analyses. † Bell 2021 used a typically developing comparison group, which does not estimate a treatment effect of the aquatic intervention; it is assessed here for transparency but is not counted among the controlled intervention studies in any domain, contributes qualitative within-group information only, and enters no GRADE judgement.

Study	Design	Tool	Selection / confounding	Intervention description	Outcome measurement	Missing data	Overall
Caputo 2018	Quasi-exp.	ROBINS-I	Some concerns: Non-random territory-based allocation; groups matched on age and ADOS but baseline CARS differed (41 vs 35)	Low risk: CI-MAT protocol clearly described with 3 phases over 40 weeks	Low risk: CARS and VABS assessed by examiners blind to group assignment	Low risk: No dropouts; all 26 participants completed	Moderate
Zanobini 2019	Quasi-exp.	ROBINS-I	Some concerns: Convenience sampling with post-hoc matching; groups from different cities (La Spezia vs Genoa)	Low risk: 7-stage protocol well described; embedded in 20-year school program	Some concerns: ABC and SRS parent-reported; parents aware of allocation; no assessor blinding	Low risk: 25/33 analyzed; 8 excluded (3 personal reasons, 5 ABC<53)	Moderate
Chu 2012	NR controlled	ROBINS-I	Some concerns: Purposive allocation to 3 groups (peer, sibling, control); non-blinded video-coded observations by graduate students involved in study	Low risk: Halliwick + TEACCH structured program clearly described	Some concerns: Observers not blinded; were graduate students involved in the study	Low risk: All 42 completed; no missing data	Moderate
Bell 2021 †	Quasi-exp. (TD comparison)	ROBINS-I	Some concerns: Non-randomized; ASD vs TD comparison (not treatment vs control); single session only; CAST used for screening (not gold-standard diagnostic)	Some concerns: Single session; limited protocol detail	Some concerns: Vagal tone measured by consumer-grade device; social checklist unvalidated	Low risk: Single session; no attrition	Moderate
Alaniz 2017	Single-group	Adapted	High risk: No comparator; N=7; maturation and regression to mean cannot be excluded	Low risk: Structured OT protocol with GAS goals	Some concerns: SSIS parent-reported; high non-compliance with questionnaires	Some concerns: Only 5/7 completed all 24 weeks	High risk
Alaniz 2020	Single-group	Adapted	High risk: No comparator; sample size not reported; probable participant overlap with Alaniz 2017 (treated as one evidence cluster)	Some concerns: Conference-supplement abstract; protocol described only in outline	Some concerns: Parent-reported measures; no assessor blinding; limited outcome detail available in abstract form	Some concerns: Attrition not reported	High risk
Alecu 2025 °	Single-group	Adapted	High risk: No comparator; N=36; concurrent ABA therapy is a major confounder	Low risk: 5-stage protocol well described	High risk: PPQ-Autism-Swim is a non-validated author-developed questionnaire	Low risk: 36/39 completed (92%)	High risk: Non-validated instrument + uncontrolled + confounded
Ennis 2011	Case series	Adapted	High risk: No comparator; N=6 completers of 11 enrolled (45.5% attrition)	Some concerns: Halliwick framework but individualised; incomplete protocol detail	Some concerns: PedsQL parent-reported; individual case format without group statistics	High risk: 5/11 (45.5%) dropout; 3 for safety/compliance reasons	High risk
Güeita-Rodríguez 2021	Single-group	Adapted	High risk: No comparator; N=6; participants had been receiving AT for ≥3 months prior (practice effect)	Low risk: WST-Halliwick with TIDieR-compliant reporting	Some concerns: Assessor was same therapist (though not involved in intervention); parent-reported PedsQL	Low risk: No dropouts; 100% attendance	High risk
Johnson 2021	Single-group	Adapted	High risk: No comparator; N=10; 3-week duration insufficient for behavioral change	Some concerns: Adapted from Alaniz; tailored to individual needs	Some concerns: Parent-reported; CBRF and PGWBI without assessor blinding	Low risk: All 10 completed all 12 sessions	High risk
Oriel 2017	Single-group	Adapted	High risk: No comparator; N=11; 4-week duration; 21.4% attrition	Some concerns: Basic framework but individualized activities	Some concerns: PDDBI parent-reported without blinding; no SDs reported	Some concerns: 3/14 (21.4%) dropout including 1 aggression episode	High risk
Oriel 2020	Single-group	Adapted	High risk: No comparator; N=13; parent/caregiver-reported	Low risk: Color-coded schedule; structured 5-stage protocol	Some concerns: HCSBS parent-reported; 2 participants had different raters at pre and post	Low risk: No dropouts; 94.2% attendance	High risk
Dowdy 2020	SCED (A-	SCED stds	Low risk: Function-based	Some concerns:	Low risk: Direct	Low risk: No	Low risk:

	B+FA)		IISCA design with clear baseline; 2 participants with replicated effects	Protocol individualized per functional analysis	behavioral observation with operationalized definitions	attrition in SCED phases	Strong SCED with functional analysis
Pushkarenko 2016	SCED (A-B-A)	SCED stds	Some concerns: 3 participants; baseline stability variable across participants	Low risk: Structured TEACCH-based activity schedules with Boardmaker pictures	Some concerns: Video coding by researcher (not independent); IOA not reported for all participants	Low risk: All 3 completed all phases	Some concerns: Limited IOA reporting
Vodáková 2022	SCED (mult. baseline)	SCED stds	Some concerns: 7 participants (1 excluded for <75% attendance); multiple baseline but staggering not fully described	Low risk: Halliwick 10-point program; individualized per skill level	Some concerns: WOTA-1 rated by same instructors who delivered intervention; not independent	Low risk: 1/8 excluded for attendance; documented	Some concerns: Assessor not independent of intervention

Supplementary Table S4. Reports not retrievable for full-text assessment (n = 35)

Full-text retrieval was attempted through institutional library searches, interlibrary-loan requests, and direct correspondence with study authors. The table lists all reports for which full text could not be obtained despite these attempts. Reports are ordered by the sequence in which they were identified during screening. Note column indicates the topic relevance to the present review and any recommended follow-up actions.

#	Year	First author / Registration ID	Title	Source	Database	Type	Note
1	2016	Lawson LM	Sensory Patterns, Obesity, and Physical Activity Participation of Children With Autism Spectrum Disorder	Am J Occup Ther	PubMed	Journal article	Non-aquatic intervention; low relevance
2	2026	Unknown	Effects of Adapted Aquatic Exercise on Behavioral Outcomes, Flexibility, and Handgrip Strength in Male Children With ASD: An RCT	ClinicalTrials.gov	Embase	Trial registration	Highly relevant; publication status unconfirmed
3	2021	Munn EE	Improvements in swim skills in children with ASD following a 5-day adapted learn-to-swim program (Ican Swim)	J Clin Med	Embase	Trial registration	Outcome focused on swim skills; moderate relevance
4	2021	Unknown	Exploration of Aquatic Therapy in Children With Autism and Their Families	ClinicalTrials.gov	Embase	Trial registration	Recommend contacting PI
5	2020	Moseley B	Group Aquatic Therapy for Children with Autism	Arch Phys Med Rehabil	Embase	Conference abstract	Possibly different study from #26
6	2020	Unknown	Effects of Outdoor Sports Activities on Pragmatic Language Skills for Children With Autism in Pakistan	ClinicalTrials.gov	Embase	Trial registration	Moderate relevance
7	2020	Thompson-Hodgetts S	A collective case study of inclusion in mainstream community programs for children with autism	Dev Med Child Neurol	Embase	Conference abstract	Recommend contacting author
8	2020	Unknown	The Impact of Group Based Activity Programmes on Children Who Have Autism and Their Families in Staffordshire	ClinicalTrials.gov	Embase	Trial registration	Possibly same project as #9
9	2019	Unknown	Keep Achieving: The Impact of Group Based Activity Programmes on Children Who Have Autism and Their Families	ClinicalTrials.gov	Embase	Trial registration	Possibly same project as #8
10	2017	Unknown	Aquatic Therapy and Autism Spectrum Disorders	ClinicalTrials.gov	Embase	Trial registration	Generic title; unconfirmed link to Moseley RCT
11	2011	Di Giacopo R	Sleep related rhythmic movements (SRMs) in two adult patients	Clin Neurophysiol	Embase	Conference abstract	Adult patients; likely not relevant
12	2010	Bernardi MM	Early immune system activation: Sexually dimorphic toxic effects of LPS on behaviour and immune parameters of rats	Toxicol Lett	Embase	Conference abstract	Animal study; not relevant
13	2021	Lapresa D	Teacher-student-task interactions in a motor skills programme for an adolescent boy with ASD: a systematic observation study	—	Web of Science	Journal article (Spanish)	Single-case observation; Spanish language
14	2017	Cei A	Motor and psychosocial development in children with ASD through soccer	—	Web of Science	Journal article	Soccer intervention; not aquatic
15	2017	Levy KM	Let's Go Under! Teaching Water Safety Skills Using a Behavioral Treatment Package	—	Web of Science	Journal article	Water safety skills; moderate relevance
16	2022	ACTRN12622001168741	Family Surfing Activity for children and adolescents with chronic conditions: effects on Health and Well-being	ANZCTR	Cochrane	Trial registration	Surfing; population not ASD-specific
17	2015	ACTRN12615001256561	Does hydrotherapy influence behaviours in children with ASD?	ANZCTR	Cochrane	Trial registration	Highly relevant; recommend priority tracking
18	2020	IRCT20180503039517N5	Effects of vitamin D and/or aquatic exercise on IL-1β and IL-1RA serum levels and behavior of children with ASD	IRCT	Cochrane	Trial registration	Aquatic exercise × ASD biomarkers; relevant
19	2021	ACTRN12621000884808	A physical activity intervention for adolescents with disability: the B2La program	ANZCTR	Cochrane	Trial registration	Non-aquatic intervention
20	2022	CTRI/2022/11/047653	Effect of Dilatory advice and exercise therapy for Autism	CTRI	Cochrane	Trial registration	Moderate relevance
21	2024	CTRI/2024/07/070209	Effects of Combined Rehabilitation Therapy for Children with ASD	CTRI	Cochrane	Trial registration	Aquatic component unconfirmed
22	2024	SLCTR/2024/023	The effect of structured physical activity on behavior, communication skills and sensory issues among children with ASD	SLCTR	Cochrane	Trial registration	Likely non-aquatic
23	2024	AdibSaber F	Comparing the influence of an aqua-based versus a mindfulness-based kata techniques training on sleep habits and stereotypic	J Pract Clin Psychol	PsycINFO	Journal article	Highly relevant; recommend priority retrieval

#	Year	First author / Registration ID	Title	Source	Database	Type	Note
			behaviors in children with autism				
24	2019	Reichle J	Considerations in implementing aided low-tech AAC systems for individuals with ASD and complex communication needs	—	PsycINFO	Journal article	AAC systems; not relevant
25	2022	Ponick C	Fisioterapia aquática no transtorno do espectro autista — TEA: estudo de caso	Rev Inspirar Mov Saude	CINAHL	Journal article (Portuguese)	Brazilian case study; relevant; Portuguese
26	2018	Moseley B	Aquatic Therapy for Treatment of ASD: A Double-Blinded, Randomized and Controlled Clinical Trial (CSM APTA 2018)	J Aquat Phys Ther	CINAHL	Conference abstract	Same Moseley RCT as #5; different conference version
27	2017	Kensinger K	The benefits of aquatic therapy as determined by a Pairwise Thematic Content Analysis of the peer-reviewed journals in therapeutic recreation	Am J Recreat Ther	CINAHL	Journal article	Secondary content analysis
28	2024	Unknown	Testing Effects of Swim Instruction on Autistic Children's Swimming and Water Safety Skills	ClinicalTrials.gov	ClinicalTrials.gov	Trial registration	Outcome focused on motor/safety skills
29	2023	Lund University	Effects of Physical Activity on Prescription (PAP) as an Individualised and Health-enhancing Intervention for Children, Adolescents and Adults With Different Disabilities	ClinicalTrials.gov	ClinicalTrials.gov	Trial registration	Multiple disabilities; ASD subgroup unconfirmed
30	2024	Oh J	Exploring the impact of parent-facilitated aquatics on children with developmental disabilities — a mixed-methods study	—	—	Journal article	Developmental disabilities (not ASD-specific)
31	2004	Carminati GG	Evolution of 2 persons with severe disability over a period of 3 years: 'Sonar Group' underwater music therapy	—	—	Journal article	Severe disability; underwater music therapy
32	2016	Chan PE	Reducing Phobic Behavior Near Water and Increasing Water Approach Skills	—	—	Journal article	Phobia reduction near water
33	1984	Killian KJ	Measuring Water Orientation And Beginner Swim Skills of Autistic Individuals	Adapt Phys Activ Q	SPORTDiscus	Journal article	Water orientation and beginner swim skills; moderate relevance
34	2014	Grosse SJ	Aquatic Safety for Individuals With Autism Spectrum Disorders	Int J Aquat Res Educ	SPORTDiscus	Journal article	Aquatic safety and drowning prevention for ASD; relevant
35	2019	Pires Pereira TL	Avaliação das variáveis comportamentais e habilidades aquáticas de autistas participantes de um programa de natação	Conexões	SPORTDiscus	Journal article (Portuguese)	Brazilian study; Portuguese language; behavioral and aquatic skills

ASD, autism spectrum disorder; ANZCTR, Australian New Zealand Clinical Trials Registry; CTRI, Clinical Trials Registry India; IRCT, Iranian Registry of Clinical Trials; SLCTR, Sri Lanka Clinical Trials Registry; AAC, augmentative and alternative communication; PI, principal investigator.

Table S5. Mapping of outcome instruments to the four pre-specified outcome domains

Instruments were assigned to domains before synthesis. Several instruments contribute to more than one domain because distinct subscales measure different constructs; in such cases only the relevant subscale was used. No instrument was used in a directly comparable form across three or more controlled studies, which is the basis for the decision not to pool. ° Alecu 2025 is listed for completeness only; it used a non-validated, author-developed instrument and contributes to no effect-direction count, GRADE judgement, or entry in Figure S1. Abbreviation expansions follow the manuscript.

Domain	Instrument (abbreviation)	Contributing studies
D1 Emotion-related functioning	Emotion Regulation Checklist (ERC); Child Behavior Checklist internalizing and anxiety subscales (CBCL); Child Behavior Rating Form (CBRF); Pervasive Developmental Disorder Behavior Inventory, arousal-related subscales (PDDBI); heart-rate variability / vagal tone; School Social Behavior Scales, hostile–irritable subscale (SSBS-2)	Marzouki 2022; Mills 2020; Johnson 2021; Oriel 2017; Ennis 2011; Bell 2021; Pan 2010; Alecu 2025 °
D2 Social-emotional functioning	School Social Behavior Scales, social competence subscale (SSBS-2); Social Skills Improvement System (SSIS); Social Responsiveness Scale (SRS); Home and Community Social Behavior Scales (HCSBS); Vineland Adaptive Behavior Scales, socialization domain (VABS); video-coded social-interaction observation	Pan 2010; Chu 2012; Zanobini 2019; Caputo 2018; Alaniz 2017; Alaniz 2020; Oriel 2017; Oriel 2020; Güeita-Rodríguez 2021; Johnson 2021; Ennis 2011; Bell 2021; Alecu 2025 °

Domain	Instrument (abbreviation)	Contributing studies
D3 Behavioral outcomes	Gilliam Autism Rating Scale (GARS-2); School Social Behavior Scales, antisocial subscale (SSBS-2); Child Behavior Checklist syndrome scales (CBCL); Aberrant Behavior Checklist (ABC); Childhood Autism Rating Scale (CARS / CARS-2); Pervasive Developmental Disorder Behavior Inventory (PDDBI); direct behavioral observation with operationalized definitions (SCEDs)	Albadry 2026; Pan 2010; Marzouki 2022; Mills 2020; Caputo 2018; Zanobini 2019; Alaniz 2017; Oriel 2017; Oriel 2020; Johnson 2021; Dowdy 2020; Pushkarenko 2016; Vodáková 2022; Alecu 2025 °
D4 Quality of life	Pediatric Quality of Life Inventory (PedsQL); Psychological General Well-Being Index (PGWBI)	Albadry 2026; Güeita-Rodríguez 2021; Ennis 2011; Johnson 2021; Alecu 2025 °
Not mapped to any pre-specified domain	Test of Gross Motor Development (TGMD-2); Water Orientation Test of Alyn (WOTA-1); Goal Attainment Scaling (GAS); Humphries' Assessment of Aquatic Readiness; PPQ-Autism-Swim (non-validated, author-developed)	Marzouki 2022; Vodáková 2022; Alaniz 2017; Pan 2010; Chu 2012; Alecu 2025 °

Methods S6. Effect-size computation

Metric. Study-level effect sizes were computed as standardized mean differences using Cohen's d: d = (M_intervention − M_comparator) / SD_pooled, where SD_pooled is the pooled within-group standard deviation. No small-sample bias correction (Hedges' adjustment) was applied: because the estimates are displayed descriptively, without pooling or inverse-variance weighting, the correction would change neither the direction nor the statistical significance of any comparison (it rescales each estimate only by a factor of about 0.95–0.98 at the sample sizes involved), and the uncorrected values remain directly comparable with those reported by the primary studies. Effect sizes were oriented so that positive values indicate improvement, regardless of the original scale direction.

Data source. Post-intervention means and standard deviations were used where reported. Change scores (pre–post differences) were used only when post-intervention values were unavailable; post-intervention and change-score metrics were never combined within the same comparison. Where standard deviations were not reported, they were derived from standard errors, confidence intervals, or exact test statistics where possible; otherwise the confidence interval was estimated from sample sizes, as indicated in Figure S1.

Conversion of reported statistics. Effects reported as t statistics were converted using $d = t \times \sqrt{(1/n1 + 1/n2)}$. *Effects reported as F statistics with one numerator degree of freedom were converted via* $t = \sqrt{F}$. *Effects reported only as exact P values were converted through the corresponding t value at the reported degrees of freedom.*

Confidence intervals. 95% confidence intervals were derived from the standard error of Cohen's d using the normal approximation: $SE(d) \approx \sqrt{[(n1 + n2) / (n1 \times n2) + d^2 / (2(n1 + n2 - 2))]}$, $CI = d \pm 1.96 \times SE(d)$.

Crossover trials. For the two crossover RCTs (Mills 2020; Pan 2010), within-participant correlation coefficients were not reported. These comparisons were therefore analysed as if they were parallel-group contrasts of post-intervention values. This approach ignores the paired design and yields conservative (wider) confidence intervals.

Uncontrolled studies. Single-group pre–post estimates were computed as within-group standardized mean changes and are displayed separately in Figure S1, labelled as uncontrolled; they were never combined with between-group estimates. Bell 2021 (typically developing comparison) and Alecu 2025 (non-validated instrument) contribute no entries to Figure S1.

Software. All computations were performed in Python 3.11.

Supplementary Appendix S1. Complete Database-Specific Search Strategies

This supplementary file documents the complete search strategies used in this systematic review. The initial search (executed 15 May 2026) used a three-concept strategy (population × intervention × outcome). In response to reviewer feedback regarding search sensitivity, the strategy was updated to a two-concept design (population × intervention only), with expanded intervention vocabulary, two additional databases, and citation tracking. The updated search was executed in July 2026 and did not identify any additional eligible studies beyond the original 19.

Changes from Original to Updated Search Strategy

Element	Original search (May 2026)	Updated search (July 2026)
Concept blocks	3 blocks: population + intervention + outcome (emotion/behavior/social-emotional/anxiety/QoL)	2 blocks: population + intervention only (no outcome restriction)
Intervention terms	aquatic, swimming, hydrotherapy, water-based, Halliwick, Watsu, aquatic exercise	Expanded: + adapted aquatics, aquatic rehabilitation, therapeutic swimming, aquatic occupational therapy, multisystem aquatic therapy, ai chi, water orientation, pool-based, pool therapy, water exercise, water therapy
Databases	6 bibliographic databases (PubMed, Embase, Web of Science, PsycINFO, CINAHL, Cochrane Library) + 1 trial registry (ClinicalTrials.gov)	8 bibliographic databases (+ SPORTDiscus, ERIC) + 1 trial registry (ClinicalTrials.gov); 9 sources in total
Citation tracking	Backward only (reference lists of included studies)	+ Forward citation tracking (Web of Science/Scopus/Google Scholar) + hand-searching of key journals + Google Scholar supplementary search
Language restriction	None	None

Search Concept Blocks

Concept Block 1 — Population (ASD)

Core free-text terms:

autis* OR "autism spectrum" OR ASD OR Asperger* OR "pervasive developmental disorder*" OR PDD OR "PDD-NOS" OR "childhood autism" OR Kanner*

Concept Block 2 — Intervention (Aquatic)

Core free-text terms:

swim* OR aquatic* OR "water-based" OR water-based OR hydrotherap* OR "pool therap*" OR "pool-based" OR Halliwick OR "water exercise*" OR "aquatic therap*" OR "aquatic intervention*" OR "aquatic exercise*" OR "aquatic rehabilitation" OR "therapeutic swim*" OR "adapted aquatic*" OR "aquatic occupational therap*" OR "multisystem aquatic therap*" OR Watsu OR "ai chi" OR "water orientation" OR "water therapy"

Note: Concept Block 3 (Outcome) was removed in the updated search to maximize sensitivity, directly addressing reviewer concerns.

Database-Specific Search Strategies

1. PubMed / MEDLINE

#1 Population:

"Autism Spectrum Disorder"[Mesh] OR "Autistic Disorder"[Mesh] OR "Asperger Syndrome"[Mesh]

OR autis*[tiab] OR "autism spectrum"[tiab] OR ASD[tiab] OR Asperger*[tiab]

OR "pervasive developmental disorder*"[tiab] OR PDD[tiab] OR PDD–NOS[tiab]

#2 Intervention:

"Swimming"[Mesh] OR "Hydrotherapy"[Mesh]

OR swim*[tiab] OR aquatic*[tiab] OR "water–based"[tiab] OR hydrotherap*[tiab]

OR "pool–based"[tiab] OR "pool therap*"[tiab] OR Halliwick[tiab]

OR "therapeutic swim*"[tiab] OR "adapted aquatic*"[tiab] OR "aquatic therap*"[tiab]

OR "aquatic intervention*"[tiab] OR "aquatic exercise*"[tiab]

OR "aquatic rehabilitation"[tiab] OR "water exercise*"[tiab]

OR Watsu[tiab] OR "ai chi"[tiab] OR "water orientation"[tiab]

OR "aquatic occupational therap*"[tiab]

#3 Final: #1 AND #2

2. Embase (Elsevier)

#1 'autism'/exp OR 'autism spectrum disorder'/exp OR 'asperger syndrome'/exp

OR autis*:ti,ab,kw OR 'autism spectrum':ti,ab,kw OR asd:ti,ab,kw

OR asperger*:ti,ab,kw OR 'pervasive developmental disorder*':ti,ab,kw

#2 'swimming'/exp OR 'hydrotherapy'/exp OR 'aquatic therapy'/exp

OR swim*:ti,ab,kw OR aquatic*:ti,ab,kw OR 'water–based':ti,ab,kw

OR hydrotherap*:ti,ab,kw OR halliwick:ti,ab,kw OR 'pool–based':ti,ab,kw

OR 'therapeutic swim*':ti,ab,kw OR 'adapted aquatic*':ti,ab,kw

OR watsu:ti,ab,kw OR 'water exercise*':ti,ab,kw

#3 #1 AND #2

3. Web of Science (Core Collection)

#1 TS=(autis* OR "autism spectrum" OR ASD OR Asperger*

OR "pervasive developmental disorder*" OR PDD–NOS)

#2 TS=(swim* OR aquatic* OR "water–based" OR hydrotherap* OR Halliwick

OR "pool–based" OR "pool therap*" OR "therapeutic swim*"

OR "adapted aquatic*" OR "aquatic therap*" OR "aquatic intervention*"

OR "aquatic exercise*" OR "water exercise*" OR Watsu OR "ai chi"

OR "water orientation")

#3 #1 AND #2

4. PsycINFO (EBSCO)

#1 (DE "Autism Spectrum Disorders" OR DE "Asperger Syndrome"
OR DE "Pervasive Developmental Disorders")
OR TI(autis* OR "autism spectrum" OR ASD OR Asperger*)
OR AB(autis* OR "autism spectrum" OR ASD OR Asperger*)

#2 (DE "Swimming" OR DE "Aquatic Locomotion" OR DE "Hydrotherapy")
OR TI(swim* OR aquatic* OR "water-based" OR hydrotherap*
OR Halliwick OR "pool-based" OR Watsu)
OR AB(swim* OR aquatic* OR "water-based" OR hydrotherap*
OR Halliwick OR "pool-based" OR Watsu)

#3 #1 AND #2

5. CINAHL (EBSCO)

#1 (MH "Autistic Disorder") OR (MH "Autism Spectrum Disorders")
OR (MH "Asperger Syndrome")
OR TI(autis* OR Asperger*) OR AB(autis* OR Asperger*)

#2 (MH "Swimming") OR (MH "Hydrotherapy")
OR TI(swim* OR aquatic* OR hydrotherap* OR Halliwick OR "water-based")
OR AB(swim* OR aquatic* OR hydrotherap* OR Halliwick OR "water-based")

#3 #1 AND #2

6. Cochrane Library (CENTRAL)

#1 MeSH descriptor: [Autism Spectrum Disorder] explode all trees

#2 (autis* OR Asperger* OR "pervasive developmental disorder*"):ti,ab,kw

#3 MeSH descriptor: [Swimming] explode all trees

#4 MeSH descriptor: [Hydrotherapy] explode all trees

#5 (swim* OR aquatic* OR hydrotherap* OR Halliwick OR "water-based" OR Watsu):ti,ab,kw

#6 (#1 OR #2) AND (#3 OR #4 OR #5)

7. SPORTDiscus (EBSCO) — NEW

#1 (DE "AUTISM") OR (DE "AUTISM spectrum disorders")
OR TI(autis* OR Asperger*) OR AB(autis* OR Asperger*)

#2 (DE "SWIMMING" OR DE "AQUATICS" OR DE "WATER exercise")
OR TI(swim* OR aquatic* OR hydrotherap* OR Halliwick OR watsu)
OR AB(swim* OR aquatic* OR hydrotherap* OR Halliwick OR watsu)

#3 #1 AND #2

8. ERIC (EBSCO) — NEW

#1 (DE "Autism Spectrum Disorders" OR DE "Pervasive Developmental Disorders")

OR TI/AB(autis* OR Asperger*)

#2 (DE "Swimming" OR DE "Aquatics")

OR TI/AB(swim* OR aquatic* OR hydrotherap* OR Halliwick)

#3 #1 AND #2

9. Trial Registries

ClinicalTrials.gov:

Condition/disease = "Autism Spectrum Disorder"

Other terms = swimming OR aquatic OR hydrotherapy OR pool

Citation Tracking and Hand-Searching

Backward citation tracking: Reference lists of all 19 included studies were screened item by item.

Forward citation tracking: All 19 included studies were searched via Web of Science, Scopus, and Google Scholar to identify all citing articles.

Hand-searching of key journals (most recent 2 years): Journal of Autism and Developmental Disorders; Research in Autism Spectrum Disorders; American Journal of Occupational Therapy; Physical & Occupational Therapy in Pediatrics; Adapted Physical Activity Quarterly.

Google Scholar supplementary search (first 200 results screened): "autism" + "swimming" / "aquatic therapy".

Search Execution and Documentation

All databases were searched within the same week (July 2026). The actual execution date was recorded for each database. Records were exported, de-duplicated (EndNote + manual review), and screened independently by two reviewers following the same protocol as the original search. The updated search confirmed that all 19 originally included studies were retrievable and that no additional eligible studies were identified.

Search records (screenshots and export files for each database) have been archived and are available upon request.
